# Open Labware: 3-D Printing Your Own Lab Equipment

**DOI:** 10.1371/journal.pbio.1002086

**Published:** 2015-03-20

**Authors:** Tom Baden, Andre Maia Chagas, Greg Gage, Timothy Marzullo, Lucia L. Prieto-Godino, Thomas Euler

**Affiliations:** 1 Werner Reichardt Centre for Integrative Neuroscience (CIN), University of Tübingen, Tübingen, Germany; 2 Bernstein Centre for Computational Neuroscience, University of Tübingen, Tübingen, Germany; 3 Institute for Ophthalmic Research, University of Tübingen, Tübingen, Germany; 4 TReND in Africa gUG, Tübingen, Germany; 5 Hertie Institute for Clinical Brain research, University of Tübingen, Tübingen, Germany; 6 Graduate School of Neural and Behavioural Sciences, International Max Planck Research School, University of Tübingen, Germany; 7 Backyard Brains, Ann Arbor, Michigan, United States of America; 8 Centre for Integrative Genomics, University of Lausanne, Lausanne, Switzerland

## Abstract

The introduction of affordable, consumer-oriented 3-D printers is a milestone in the current “maker movement,” which has been heralded as the next industrial revolution. Combined with free and open sharing of detailed design blueprints and accessible development tools, rapid prototypes of complex products can now be assembled in one’s own garage—a game-changer reminiscent of the early days of personal computing. At the same time, 3-D printing has also allowed the scientific and engineering community to build the “little things” that help a lab get up and running much faster and easier than ever before.

## Abstract 2

Free-and-open online designs for self-built, sophisticated lab equipment provide access to teaching and research tools for an increasingly broad audience worldwide.

Applications of 3-D printing technologies (Fig. 1A, Box 1) have become as diverse as the types of materials that can be used for printing. Replacement parts at the International Space Station may be printed in orbit from durable plastics or metals, while back on Earth the food industry is starting to explore the same basic technology to fold strings of chocolate into custom-shaped confectionary. Also, consumer-oriented laser-cutting technology makes it very easy to cut raw materials such as sheets of plywood, acrylic, or aluminum into complex shapes within seconds. The range of possibilities comes to light when those mechanical parts are combined with off-the-shelf electronics, low-cost microcontrollers like Arduino boards [1], and single-board computers such as a Beagleboard [2] or a Raspberry Pi [3]. After an initial investment of typically less than a thousand dollars (e.g., to set-up a 3-D printer), the only other materials needed to build virtually anything include a few hundred grams of plastic (approximately US$30/kg), cables, and basic electronic components [4,5].

**Fig 1:**
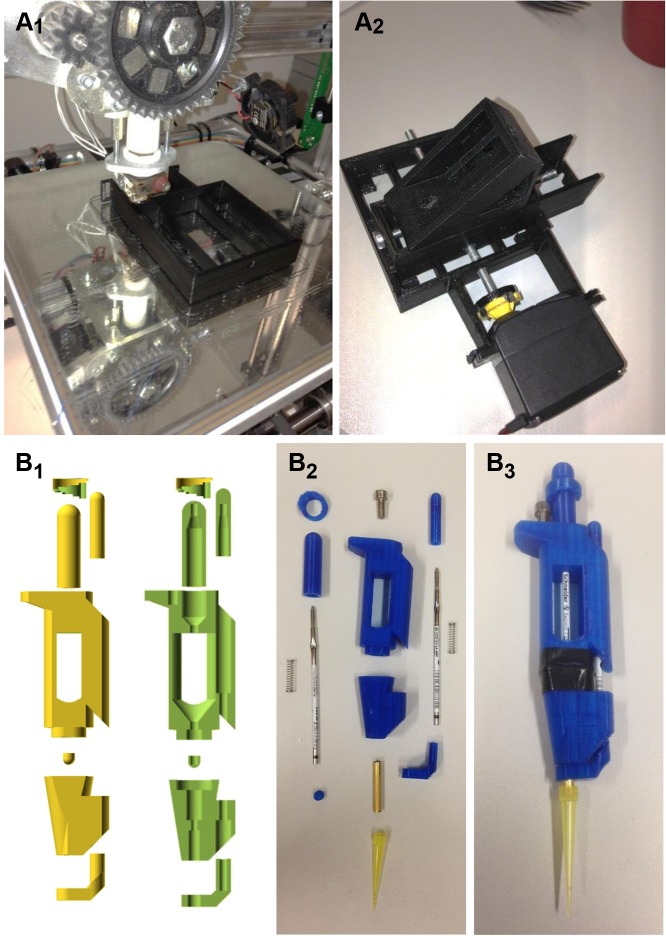
Examples of open 3-D printed laboratory tools. **A**_**1**_, Components for laboratory tools, such as the base for a micromanipulator [18] shown here, can be rapidly prototyped using 3-D printing. **A**_**2**,_ The printed parts can be easily combined with an off-the-shelf continuous rotation servo-motor (bottom) to motorize the main axis. **B**_**1**_, A 3-D printable micropipette [8], designed in OpenSCAD [19], shown in full (left) and cross-section (right). **B**_**2**_, The pipette consists of the printed parts (blue), two biro fillings with the spring, an off-the-shelf piece of tubing to fit the tip, and one screw used as a spacer. **B**_**3**_, Assembly is complete with a laboratory glove or balloon spanned between the two main printed parts and sealed with tape to create an airtight bottom chamber continuous with the pipette tip. Accuracy is ±2–10 μl depending on printer precision, and total capacity of the system is easily adjusted using two variables listed in the source code, or accessed via the “Customizer” plugin on the thingiverse link [8]. See also the first table.

**Box 1. Glossary**

**Open source**

A collective license that defines terms of free availability and redistribution of published source material. Terms include free and unrestricted distribution, as well as full access to source code/blueprints/circuit board designs and derived works. For details, see http://opensource.org.

**Maker movement**

Technology-oriented extension of the traditional “Do-it-Yourself (DIY)” movement, typically denoting specific pursuits in electronics, CNC (computer numerical control) tools such as mills and laser cutters, as well as 3-D printing and related technologies.

**3-D printing**

Technology to generate three-dimensional objects from raw materials based on computer models. Most consumer-oriented 3-D printers print in plastic by locally melting a strand of raw material at the tip (“hot-end”) and “drawing” a 3-D object in layers. Plastic materials include Acrylnitrile butadiene styrene (ABS) and Polylactic acid (PLA). Many variations of 3-D printers exist, including those based on laser-polymerization or fusion of resins or powdered raw materials (e.g., metal or ceramic printers).

**Arduino boards**

Inexpensive and consumer-oriented microcontroller boards built around simple processors. These boards offer a variety of interfaces (serial ports, I2C and CAN bus, etc.), μs-timers, and multiple general-purpose input-output (GPIO) pins suitable for running simple, time-precise programs to control custom-built electronics.

**Single board computers**

Inexpensive single-board computers capable of running a mature operating system with graphical-user interface, such as Linux. Like microcontroller boards, they offer a variety of hardware interfaces and GPIO pins to control custom-built electronics.

It therefore comes as no surprise that these technologies are also routinely used by research scientists and, especially, educators aiming to customize existing lab equipment or even build sophisticated lab equipment from scratch for a mere fraction of what commercial alternatives cost [6]. Designs for such “Open Labware” include simple mechanical adaptors [7], micropipettes (Fig. 1B) [8], and an egg-whisk–based centrifuge [9] as well as more sophisticated equipment such as an extracellular amplifier for neurophysiological experiments [10], a thermocycler for PCR [11], or a two-photon microscope [12]. At the same time, conceptually related approaches are also being pursued in chemistry [13–15] and material sciences [16,17]. See also Table 1.

**Table 1:** Open Labware designs for a biology lab.

Area	Project	Source
Microscopy	Smartphone Microscope	http://www.instructables.com/id/10-Smartphone-to-digital-microscope-conversion
	iPad Microscope	http://www.thingiverse.com/thing:31632
	Raspberry Pi Microscope	http://www.thingiverse.com/thing:385308
	Foldscope	http://www.foldscope.com/
Molecular Biology	Thermocycler (PCR)	http://openpcr.org/
	Water bath	http://blog.labfab.cc/?p=47
	Centrifuge	http://www.thingiverse.com/thing:151406
	Dremelfuge	http://www.thingiverse.com/thing:1483
	Colorometer	http://www.thingiverse.com/thing:73910
	Micropipette	http://www.thingiverse.com/thing:255519
	Gel Comb	http://www.thingiverse.com/thing:352873
	Hot Plate	http://www.instructables.com/id/Programmable-Temperature-Controller-Hot-Plate/
	Magnetic Stirrer	http://www.instructables.com/id/How-to-Build-a-Magnetic-Stirrer/
Electrophysiology	Waveform Generator	http://www.instructables.com/id/Arduino-Waveform-Generator/
	Open EEG	https://www.olimex.com/Products/EEG/OpenEEG/
	Mobile ECG	http://mobilecg.hu/
	Extracellular amplifier	https://backyardbrains.com/products/spikerBox
	Micromanipulator	http://www.thingiverse.com/thing:239105
	Open Ephys	http://open-ephys.org/
Other	Syringe pump	http://www.thingiverse.com/thing:210756
	Translational Stage	http://www.thingiverse.com/thing:144838
	Vacuum pump	http://www.instructables.com/id/The-simplest-vacuum-pump-in-the-world/
	Skinner Box	http://www.kscottz.com/open-skinner-box-pycon-2014/

See also S1 Data.

## A Culture of Sharing

Most makers share their designs under an open source license together with detailed assembly instructions in online repositories [20–22] such as the National Institutes of Health (NIH) 3-D print exchange [23] or in peer-reviewed journals [10,17,24–29]. As a result, anyone can freely use and modify them. This open culture, which has transformed the world of software engineering over the past decades, offers several advantages over traditional product design. First, designs are not only free, but are directly shaped by people who will actually use the final product. Second, building your own experimental equipment yields a much deeper understanding of the principles underlying its design and a better awareness of its limits. Third, manufacturing is immediate and local—thus empowering laboratories and schools located in difficult-to-reach places. Fourth, the open source movement is a global phenomenon, connecting people worldwide, often to recruit talented builders and coders from outside the traditional scientific establishment. Still, there are drawbacks. For example, a commercial solution may be preferred if the time and cost of in-house development exceeds the benefits of control and instrument knowledge. Nevertheless, and perhaps counter-intuitively, some open designs are published by commercial companies that offer the parts as well as the assembled product with technical support for a fee, while maintaining the source material online under an open source license. Consumers can more freely balance cost against time to build, while companies gain in customer relations and product feedback.

## Quality Control and the Evolution of Open Labware Designs

**Fig 2:**
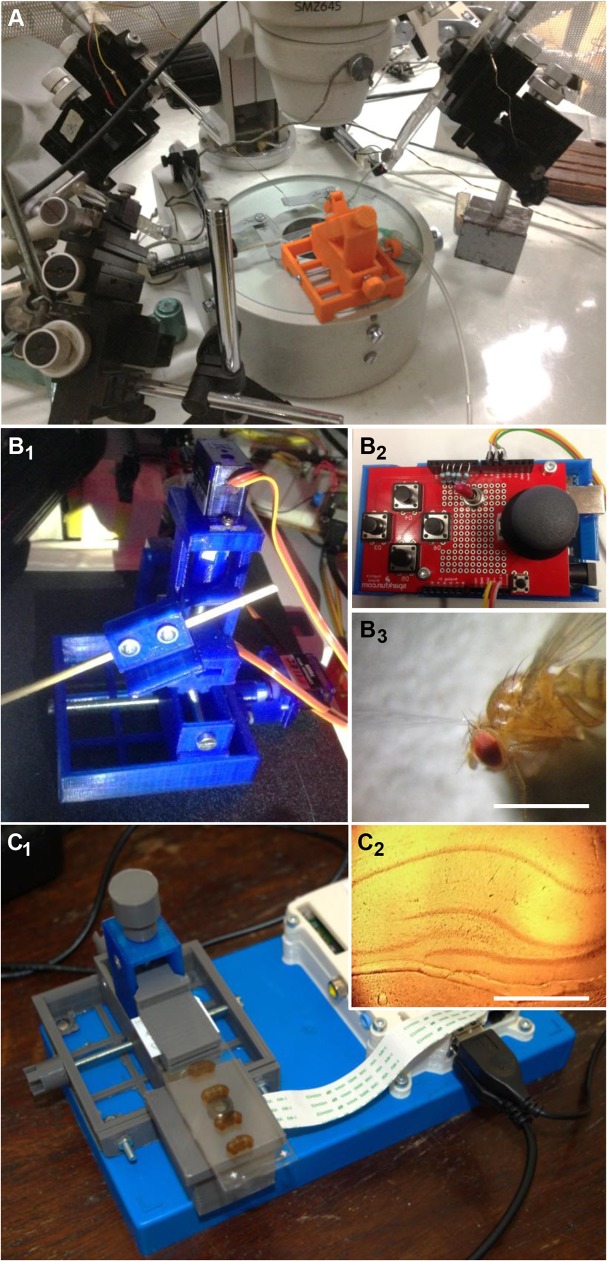
Evolution of an Open Labware design. **A**, A 3-D printable micromanipulator with a slanted Z-axis [18], here shown amidst commercial alternatives, initially served as the basis for a motorized version with “real” Z axis [32] (B). **B**_**1,2,**_ the three axes are driven by continuous-rotation micro-servos, controlled by an Arduino fitted with a Joystick-shield and a 9V battery. **B**_**3**_, The motorized manipulator offers sufficient precision to target individual hairs on the head of a fruitfly (±5–20 μm during movements, depending on printer precision; <1 μm drift min^-1^ when stationary). Scale bar 1 mm. **C**_**1**_, The same manipulator build was then converted into a microscope-stage to permit accurate placement and focus of histology samples [34]. The optics are provided by an off-the-shelf, low-power acrylic lens positioned directly above a Raspberry Pi camera module [3]. C_2_, Image taken with the microscope, showing a slice of mouse brain (hippocampus) stained for cytochrome oxidase C. Scale bar 500 μm.

The flagship of the open source movement, the operation system GNU/Linux [30], is perhaps the best example for the potential of open designs. First available in the 1990s, today it is one of the most widely used system on supercomputers, servers, and mobile phones (Android) and increases its share of home users every year. Critically, Linux is fully open source—anyone can freely access its entire source code, change it, and distribute modifications. Its success flows from the idea that “given enough eyeballs all bugs are shallow” [31]. In other words, a distributed network of collaborators helps to identify and rectify bugs in the system as they arise. Open Labware works the same way: release of a design sparks feedback and refinements from the community. Although Open Labware’s contributor base is still relatively small, feedback can nonetheless markedly improve product design and expand potential applications over time. For example, free online sharing of the designs for a manually controlled 3-D printed micromanipulator (Fig. 2A) [18] provided the starting point for a high-precision motorized version (Fig. 2B) [32]. Subsequently, and in combination with open designs for smartphone lens-adapters offering portable options for field-microscopy [33], both designs led to the “Raspberry Pi-scope” [34]—a self-standing histology microscope based on a Raspberry Pi, equipped with a high-resolution camera module, a low-cost acrylic lens, and the manipulator body to accurately position the sample (Fig. 2C). One next step in this evolution may be to add low-cost fluorescence capability through the addition of a ultraviolet light-emitting diode (UV-LED) and the appropriate filters, perhaps inspired by materials used in the less than US$1 fluorescence-capable Foldscope [24]. Without free-and-open sharing of the complete source materials at all stages of development, this evolution would not have been possible. With educators and researchers increasingly integrating Open Labware approaches into their projects, designs are expected to continuously improve and diversify.

## Online Resources for Learning and Problem Solving

Although most Open Labware designs are published with detailed assembly instructions aimed at the non-specialist, a basic understanding of concepts in physics, electronics, and computer programming is certainly helpful. Luckily, freely available online resources facilitate self-learning (Table 2). In addition, many makers hone their skills directly at the workbench, simply by attempting to recreate or modify existing designs. Expert community help can be rapidly found in online forums such as Stack Overflow [35], returning to the idea that many eyes lead to rapid problem-solving. In addition, online aggregators gather and summarize information on specific topics, serving as a hub for both information seekers and distributors. Some of them are curated in a centralized manner [36], while others follow the distributed wiki principle [37].

**Table 2:** Open resources for self-taught learning of programming, electronics, and basic physics.

Project	Description	Source
Code.org	Well-structured teaching units to learn computer programming, mainly aimed at beginners.	http://code.org/
Code Academy	Teaches a wide range of programming languages based on brief explanations followed by exercise-based learning.	http://codeacademy.com
Khan Academy	Promotes self-paced information technology (IT) education for high school curricula and beyond through videos and exercises.	http://khanacademy.org
Software carpentry	Aimed at scientists to improve coding and data management skills through online material, as well as offline lectures and bootcamps.	http://software-carpentry.org/
Sparkfun tutorials	Company selling electronic components—their website contains a free tutorial section teaching basic electronics.	https://learn.sparkfun.com/tutorials
Openeuroscience	Gathers open source projects for neurosciences.	http://openeuroscience.wordpress.com
Labrigger	Gathers both open and closed source software and hardware projects for biomedical research.	http://labrigger.com
Hackteria	Website that gathers open source projects for DIY biology	http://hackteria.org/wiki/index.php
Appropedia	Wiki page that gathers information on environmental, sustainability-related projects.	http://www.appropedia.org/
Openstax college	Non-profit foundation creating open college-level textbooks (e-books).	http://www.openstaxcollege.org

## Application in a Resource-Challenged Context

**Fig 3:**
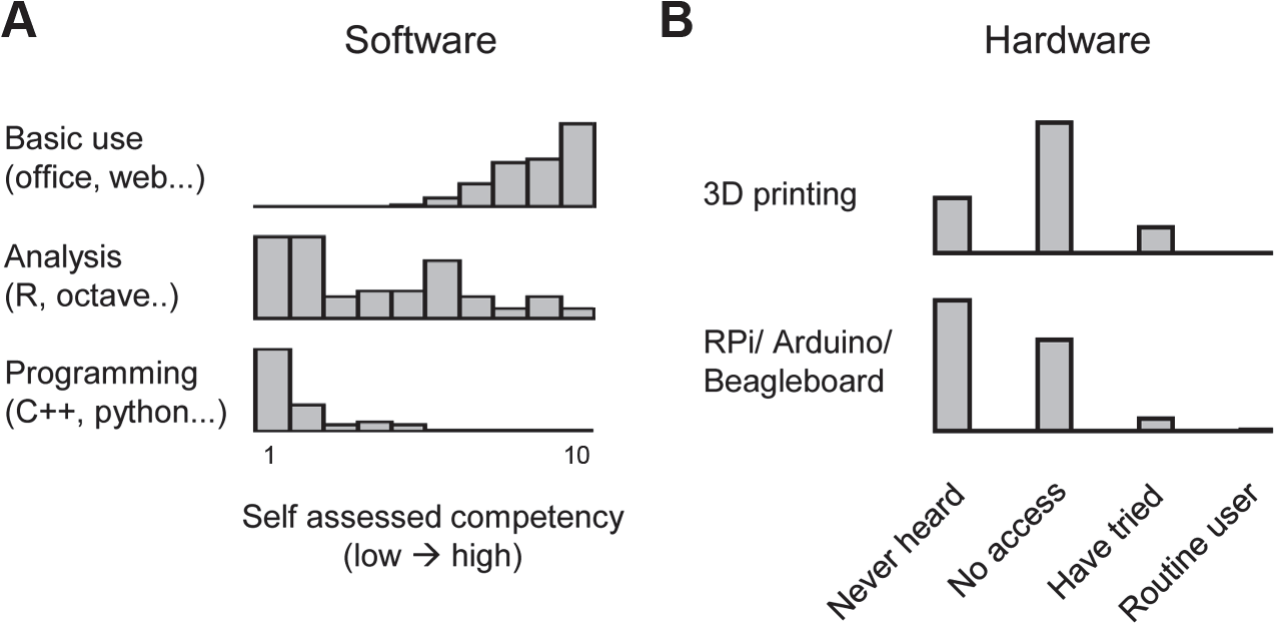
Open Labware at universities in sub-Saharan Africa. An online survey was taken by 89 biomedical researchers (MSc. to Professor) at universities in 12 different sub-Saharan African countries in August 2014. Researchers rated their own competency and awareness in aspects of software and hardware usage. **A**, Software competency rated on a scale of 1 (low) to 10 (high) in “basic usage such as navigating office software or the internet,” “usage of open analysis packages such as R [44], octave [45], or similar,” and “programming, e.g., using C++, python, or any other mainstream language.” **B**, Hardware awareness for possibilities in “3-D printing” (top) and “single board computers/microcontrollers such as Raspberry Pi, Arduino, Beagleboard, or similar” rated in four categories: (i) “I have never heard of this,” (ii) “I have heard of it but I have no access,” (iii) “I have tried using this at least once,” (iv) “I am a competent/routine user.”

If a commercial alternative is available, it is usually the compromise between time and money spent that determines whether to build or purchase equipment. While Open Labware designs may benefit any research or education setting [38], one obvious foothold for their possibilities lies in economically deprived schools and universities in the developing world. Here, the introduction of Open Labware may make the crucial difference between having some usable equipment to work with and having none at all. Several benefits of an open model come to light: low cost, local manufacture, and the possibility to customize according to local demands or availability of parts. In sub-Saharan Africa, only a few bases and organizations that offer training in “maker” skills or general promotion of open source principles operate. These include the Free Software and Open Source Foundation for Africa (FOSSFA [39]), Fundi bots [40], as well as a few physical spaces such as FabLabs [41] and Maker- [42] and Hackerspaces [43] scattered mostly around the major cities. In addition, companies selling easily transportable 3-D printers and supplies are rapidly establishing their foothold on the African continent, such that local sourcing of the required hardware is no longer an insurmountable obstacle. However, on the whole, Open Labware possibilities remain poorly established in this context, as attested in a survey taken by 89 biomedical researchers from 12 sub-Saharan African countries in August 2014 (Fig. 3). When asked about their software competency, most respondents indicated that while they are comfortable with “basic” IT usage (defined as using office packages or navigating the web), few were routine users of standard open analysis packages such as R [44], octave [45], or other Python [46] based packages, and programming skills were even less prevalent (Fig. 3A). Awareness and competency in the use of open hardware approaches were even less developed: over 90% of respondents (83/89) had never used 3-D printing or any form of single-board computers or microcontrollers (Fig. 3B).

As part of ongoing efforts to promote scientific education and research in the developing world, we introduced the many possibilities of Open Labware to schools and universities in sub-Saharan Africa (TReND in Africa [47]) and Latin America (Backyard Brains [48]). Different formats were used, ranging from three-week intensive neuroscience summer schools aimed at university graduates in Uganda and Tanzania (four events), to multiday MakerSpace workshops aimed at school students and their teachers in Chile and Mexico (12 events), to outreach events and lectures held at scientific conferences, schools, and public spaces on four continents (more than 100 events). Here, we present some experiences and success stories from this work.

## In-Depth Exposure during Multiday Training Events

**Fig 4:**
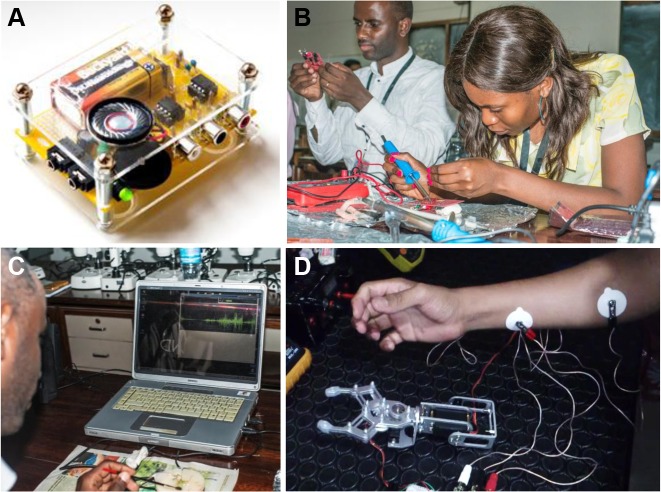
Hands-on exposure to Open Labware in the developing world. **A**, Each student assembled a spikerbox [10,49], an amplifier for neurophysiological experiments, from its off-the-shelf components. **B**, Students at a workshop in Dar es Salaam, Tanzania. **C**, The assembled amplifiers were subsequently used to perform simple neurophysiological experiments [51]. Image credit for panels B and C: Horst Schneider. **D**, One student set up a spikerbox via an Arduino to trigger closure of a “gripper-hand,” a low-cost robotic limb, through contractions of their forearm muscles.

The longer events, such as the three-week neuroscience summer schools in Africa or the multiday MakerSpace workshops in Latin America, afforded participants the opportunity to get hands-on experience using, assembling, and contributing to the development of Open Labware designs. One popular activity was to assemble existing designs (such as a spikerbox, Fig. 4A [10,49]) from off-the-shelf parts. This required participants to study the circuit diagram, note the identity and polarity of simple electronic components such as chips and capacitors and to solder them in place on the printed circuit board (PCB) (Fig. 4B). The assembled amplifiers were subsequently used to perform classic neurophysiological experiments such as recording of action potentials from the locust [50] or cricket [51,52] extensor tibiae (Fig. 4C). Although initially daunting especially for the many participants without previous contact with any form of electrical engineering, the experience tended to be very motivating across ages and cultures. In particular, it contributed to take away the fear of experimenting with simple electronic circuits or opening up and attempting to modify or repair existing electronic tools in their daily environment. The low cost of required parts also allowed participants to build their own gear, rather than that of their school or university, making them more invested in its success and maintenance. Critically, building equipment from scratch often resulted in a high level of mechanistic understanding and a curiosity to “play” with the finished product to see if it can be improved or modified to better suit a particular purpose. For example, one undergraduate student linked an electromyogram (EMG)-amplifier to an off-the-shelf “robotic limb” [53] via an Arduino microcontroller [1] to remotely control grasping movements by contracting their forearm muscles (Fig. 4D). Clearly, introduction of low-cost and open source electronics and mechanical parts has the potential to open up vast possibilities to resourceful people anywhere in the world, independent of financial means or educational background.

During the three-week neuroscience summer schools in Uganda and Tanzania [54] we introduced participants to 3-D printed lab tools such as pipettes [8], manipulators [18,32], and microscope adapters [55] and compared the usefulness of self-built versus commercially available solutions for different types of experiments. For example, although printed pipettes [8] offer lower precision than commercially available ones (±2–10 μl, depending on printer precision) they are nonetheless adequate for many “low precision” tasks such as distributing diluted antibody solutions to different samples or applying mounting media for immunohistochemistry. Similarly, we used different versions of 3-D printed micromanipulators [18,32] to position reference electrodes during neurophysiological experiments, e.g., for *Calliphora* tangential cell recordings [56], but maintained the recording tungsten electrode on a more stable commercial manipulator. For some types of recordings, such as *Drosophila* electroretinograms (ERGs) [57], the printed manipulators were adequate to hold both electrodes, and the magnification gained by attaching a low-power acrylic lens to a webcam or smartphone easily sufficed for their accurate placement. We also provided access to pre-installed Raspberry Pi computers [3] running a range of open analysis and office software packages and provided basic training in their use. Hands-on exposure allowed students to judge for themselves which designs would be useful in their own research and teaching activities. In a subsequent survey, students rated the usefulness of every open design described above as at least nine out of ten on average (*n* = 33). We believe that the introduction of Open Labware possibilities to the African university system may be a highly effective measure towards fostering excellence in research and education on the continent.

## Motivating More High School Graduates to Pursue a Career in Science

One pervasive issue in building a sustainable research infrastructure and scientific culture in resource-challenged countries is a perceived limit on career choices afforded by higher education. Traditionally, three disciplines—medicine, civil engineering, or law—are considered the best choices for reliable income, with few individuals enrolling in a natural science subject and fewer still ending up in active research [58,59]. To encourage students to consider a career in science, we (TReND and Backyard Brains) have participated in more than a hundred science outreach events around (Neuro)science, engaging more than 10,000 students, parents, and teachers. In several science camps (“ChileVA! [60]”), we gave two-hour lectures to high school students about the brain and how to record from neurons and muscles. The students not only “tolerated” this unusually long-format science lecture without breaks, but swarmed the demonstration booth afterwards. Clearly, low-cost and portable equipment offers the possibility to perform science demonstrations anywhere, independent of local infrastructure.

In TReND’s African activities, student-alumni of our summer workshops have taken over local science outreach by organizing into regional teams to visit schools and universities in their respective home countries [61]. Many of these young scientists come from similar backgrounds as the students they engage, allowing them to act as powerful role models. Thus, students attending a school that cannot afford the equipment or infrastructure to perform live experiments during science classes are exposed to local scientists with a similar background, handling equipment they know how to build from affordable, local resources. The experience can be very powerful and inspire them to pursue a similar career, as well as offering local teachers ideas to use in future science classes. This approach of “teaching the teachers” also means that the impact of educating a few students at a high level has the potential to trickle-down and achieve a wide impact in the long term.

## Policy Recommendations

Clearly, the use and design of Open Labware designs can be a powerful ingredient to foster scientific research, education, and public science engagement. Their evolution spans several disciplines, from computer sciences and mechanical engineering to electronics and biology—thus connecting experts and the wider public across fields and sparking creativity in people of all ages. Their low cost, adaptability and robustness renders designs suitable for a broad range of applications in both teaching and research. Below we present some suggestions for policy implementations to optimize available possibilities.

1. Integrate more aspects of design and use of Open Labware into traditional science curricula, ideally at an early age.
2. Establish more hands-on training courses in basic hardware design and programming skills for established scientists and educators.
3. Establish infrastructural support to afford more students and educators easy and direct access to 3-D printing and related technologies. With 3-D printers starting at a few hundred dollars and their price steadily falling, schools and university departments should not be barred from investing in their own model because of economic reasons.
4. Provide incentives for companies to invest in an open model of product design. This move promises to spark a new generation of open companies in more direct dialogue with the end user, towards better, individually tailored, and more affordable product design.

## Supporting Information

S1 Data Thingiverse files. Container with all files mentioned in the article that are currently hosted on thingiverse (www.thingiverse.com).
(ZIP)

## Abbreviations:

ABS: Acrylnitrile butadiene styrene
CNC: computer numerical control
DIY: Do It Yourself
EMG: Electromyogram
ERG: Electroretinogram
FOSSFA: Free Software and Open Source Foundation for Africa
GNU: “GNU is not Unix” (recursive acronym)
GPIO: general-purpose input-output
IT: information technology
LED: Light Emitting Diode
NIH: National Institutes of Health
PCB: Printed Circuit Board
PLA: Polylactic acid
TReND: Teaching and Research in Natural Sciences for Development (in Africa)
UV: Ultraviolet
