# Correction: Open Labware: 3-D Printing Your Own Lab Equipment

**DOI:** 10.1371/journal.pbio.1002175

**Published:** 2015-05-21

**Authors:** Tom Baden, Andre Maia Chagas, Gregory J. Gage, Timothy C. Marzullo, Lucia L. Prieto-Godino, Thomas Euler

The names of the 3^rd^ and 4^th^ authors are incorrect and should be: Gregory J. Gage and Timothy C. Marzullo, respectively. Please use the corrected citation below.

‘The NIH 3-D print exchange’ was referenced incorrectly in the text. The correct version is: The NIH 3D Print Exchange. For this reason, Reference 23 is incorrect and should be: National Institutes of Health (2015) The NIH 3D Print Exchange. http://3dprint.nih.gov/.
