# Supplementary material for: Open Labware: 3-D Printing Your Own Lab Equipment
Source: PLoS Biol. 2015 Mar 20;13(3):e1002086. doi: 10.1371/journal.pbio.1002086 (PMC4368627; doi:10.1371/journal.pbio.1002086)
Supplement: S1 Data — Container with all files mentioned in the article that are currently hosted on thingiverse (www.thingiverse.com). (ZIP) [file pbio.1002086.s001.zip › Biropette_customisable_high_precision_pipette - by T Baden.zip!/Biropette_customisable_high_precision_pipette/Pipette_v15.pdf]

Biropette: 3D printed, customizable high precision pipette

2014 Feb 23, pipette v15

Tom Baden
Centre for Integrative Neuroscience (CIN)
University of Tübingen, Germany

thomas.baden@uni-tuebingen.de
Thingiverse ID: tbaden
Personal website: http://tombaden.wordpress.com/

Science in Africa NGO
TReND in Africa gUG: http://trendinafrica.org/

Help us design cheap, good lab equipment!
http://trendinafrica.org/activities/open-source/open-source-main/

We want your brain!
(in a completely non-zombie fashion)

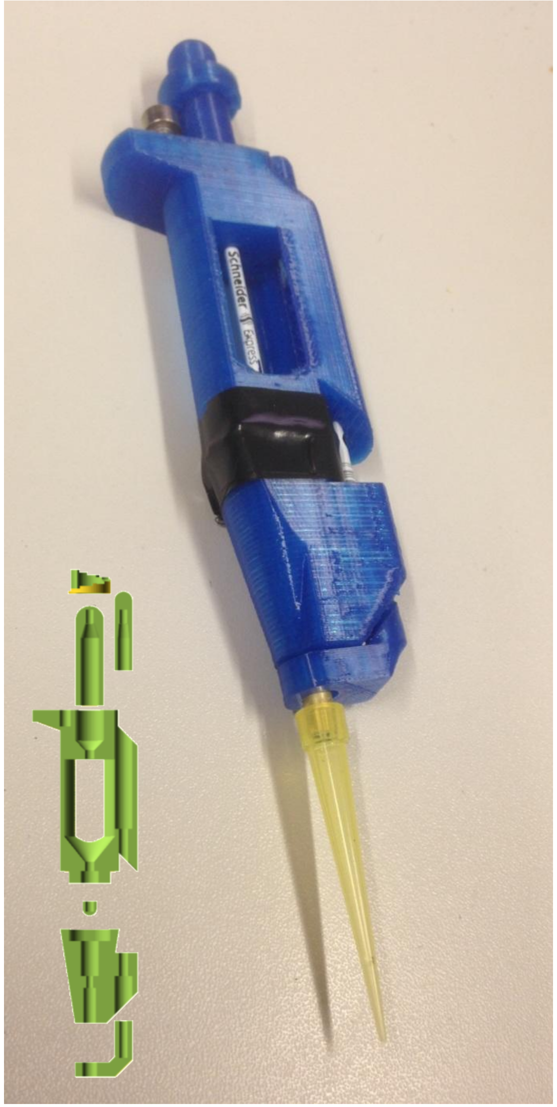

*inspired by the “Straw pipette“ (http://www.thingiverse.com/thing:64977)
by Konrad Walus (kwalus),
University of British Columbia, Vancouver, CA

Overview

Vanilla version
“expunger = 0“

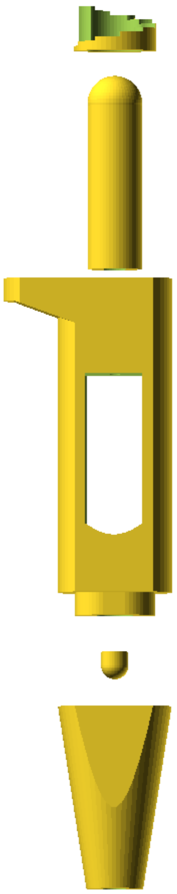

Full version

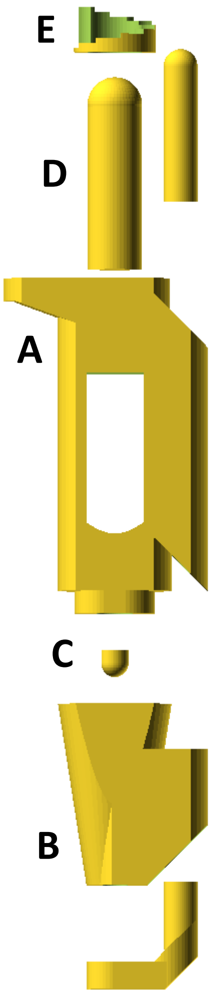

Cross-section
“cross-section = 1“

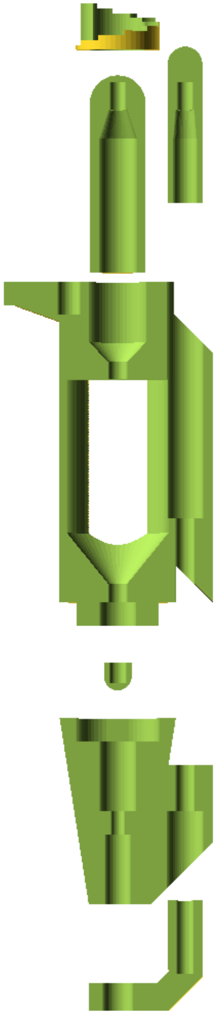

Printed on Felix printer
normal quality, no supports: ~3 hrs

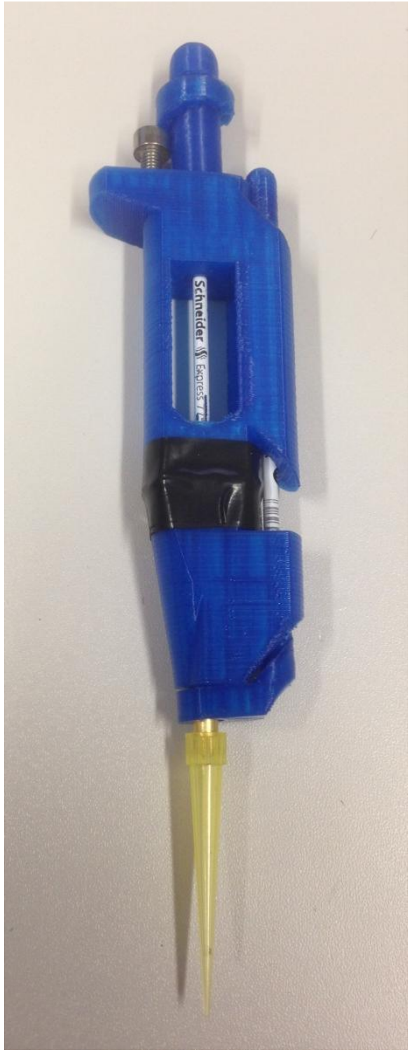

Part list

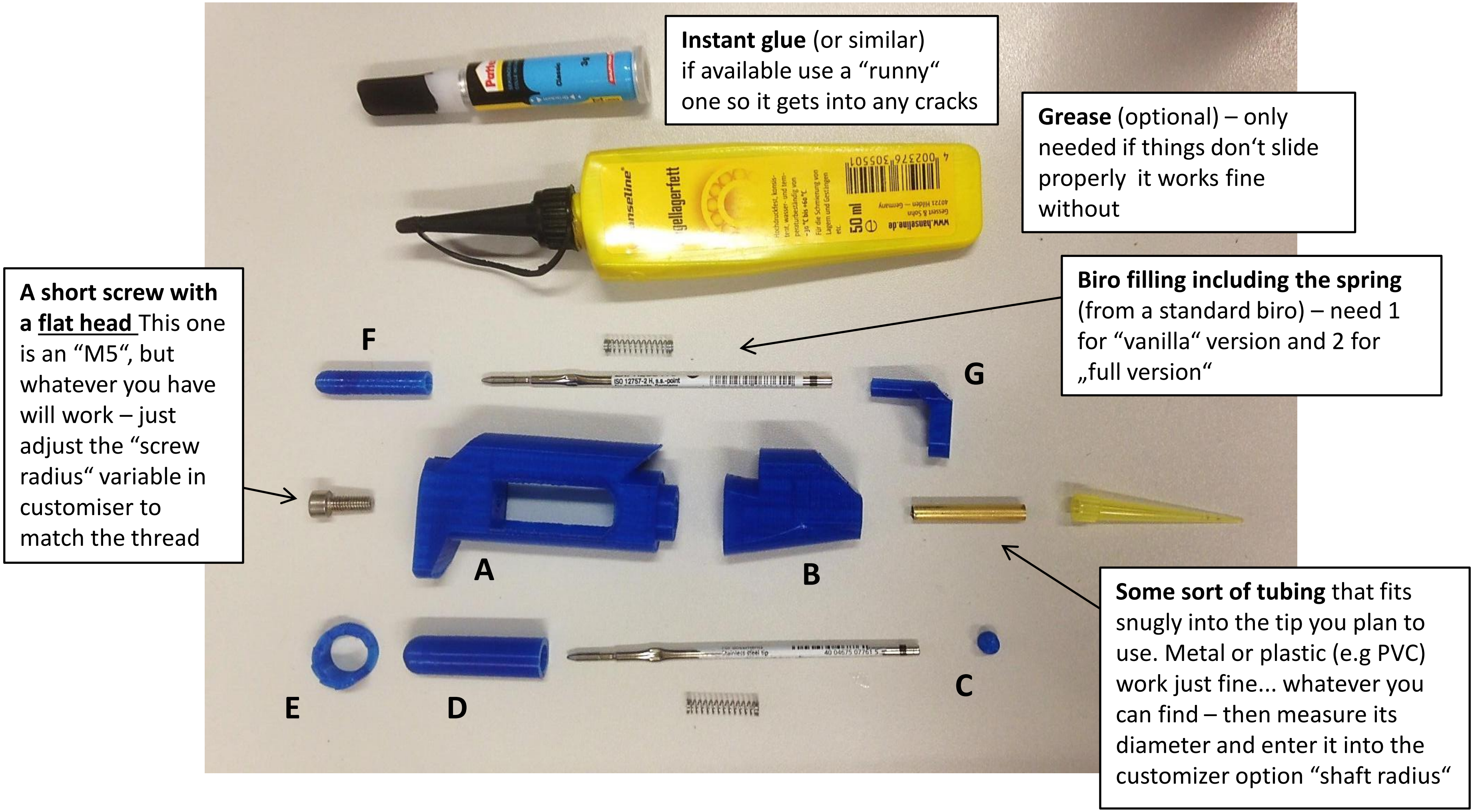

You will also need something to use as membrane!
(e.g. Lab glove, baloon, condom... anything that is strong and flexible should do)
plus some descent tape and/or Parafilm

Some quick and dirty calculations

Volume stepper
By default it is 10 mm tall and has 10 steps, so 1 mm displacement per step. This is customizable under “Range Z“ and “Range Steps“

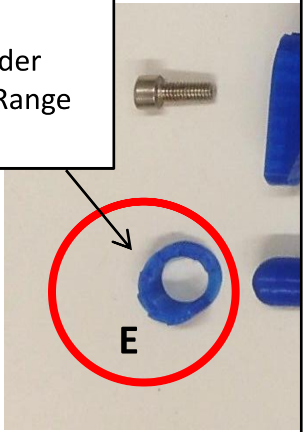

This tiny thing is key to determine the volume range of the system.

It sits on the bottom of the main axis and displaces the membrane. A bigger radius part here will displace the membrane more for an equal displacement in Z (customizer variable “range membrane pin“). You can calculate the total range of the pipette (approximately) as follows.

Total volume: V_T
Radius of “C“: r
Button Range: Z (RangeZ)

$$V_T = Z * \pi * r^2$$

and the Volume per step (V_s),
Number of Steps: S

$$V_S = Z * \pi * r^2 / S$$

So in default settings:
r = 3 mm = $3 * 10^{-3}$ m
Z = 10 mm = 10^{-2} m
S = 10 steps

$$V_T = 10^{-2}\ m * 3.141 * 9.\ 10^{-6}\ m^2$$
$$= \sim 2.83 * 10^{-4}\ m^3$$
$$= \sim 283\ \mu l$$

and

$$V_S = 28.3\ \mu l$$

Note that this is an overestimate – really you only have 8 steps as the last is used as a stopper, and the 1st is the base. Plus this calculation ignores that the semi-sphere of part C (i.e. its head) may not perfectly protrude into the membrane at rest (Volume of a sphere = $4/3\ \pi * r^3$). So multiply all by 0.8 and subtract a bit for the sphere thing... Finally, the membrane forms a bit of a tent... Overall, this gives roughly 200 µl. You can calibrate your pipette quickly when done (e.g. by using high precision scales and distilled water; 1 mg = 1 µl). If you don‘t have high precision scales, use low precision ones and pipette everything e.g. 10 or 100 times to get into range and rescale accordingly... Note that the “steps“ are going to be quite linear so no need to properly calibrate them all... Just divide V_T by (nSteps-2)

$$V_T\ (actual) = \sim 200\ \mu l$$

Tip: Just type it into google!

“10mm * pi * (3mm)^2“

It conveniently gives the answer in milliliters, so you dont have to worry about making log unit errors!

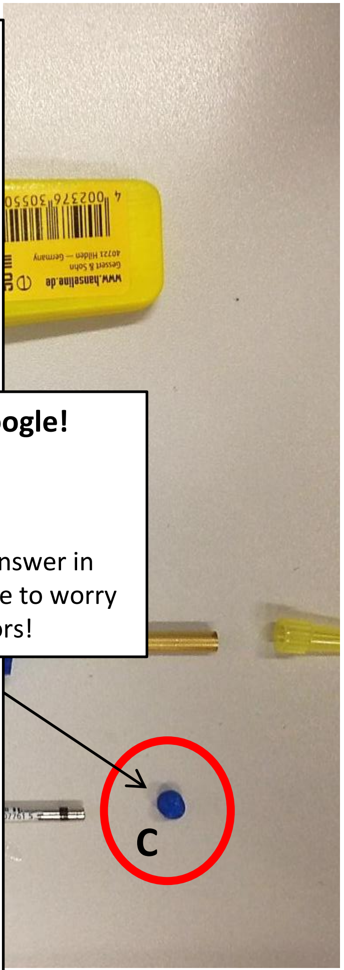

Assembly 1/5

Make sure your piece of "tubing" fits snug into part B.

The length should be approx as illustrated, such that when the tip is mounted, there are a few mm space between part G and the tip

When happy, superglue the tube in place (without part G, obviousy)

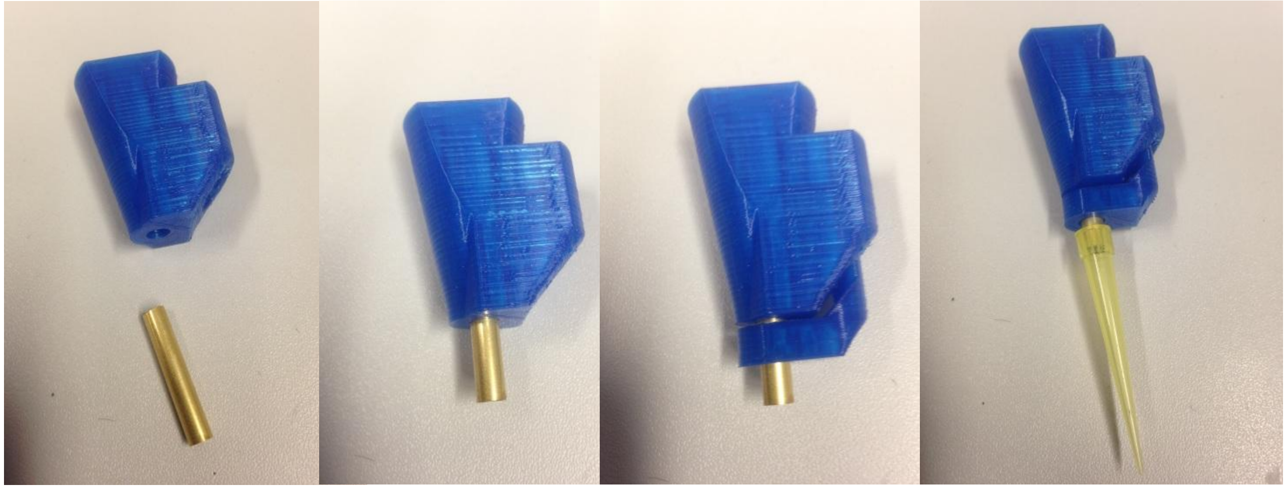

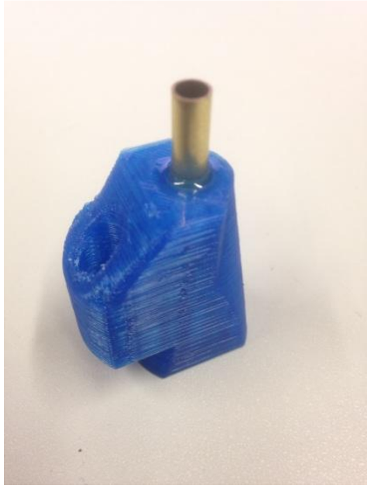

Use copious amounts of glue to seal any escapes for air!

Key: part B is the only one that needs to be properly airtight. So don't skimp on the print settings on this one. If your pipette is leaky, it almost certainly is due to a leaky part B. I actually found that the more "oozy" Vellemann K8200 prints work better than the more precise Felix prints. I guess the PLA really fills any cracks if a tad oozy. (shown here is a Felix one)

Assembly 2/5

Slip on part C on the bottom. It should protude a tad. If not, slip something (e.g. a washer) under it so that it does.

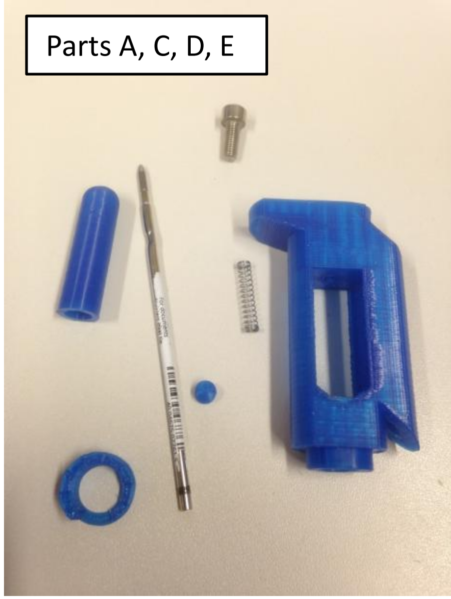

Parts A, C, D, E

Put the spring onto the biro filling the wrong way round

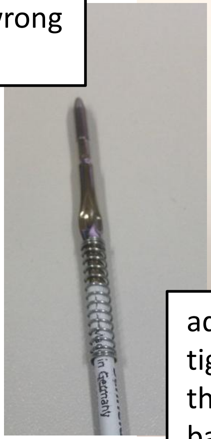

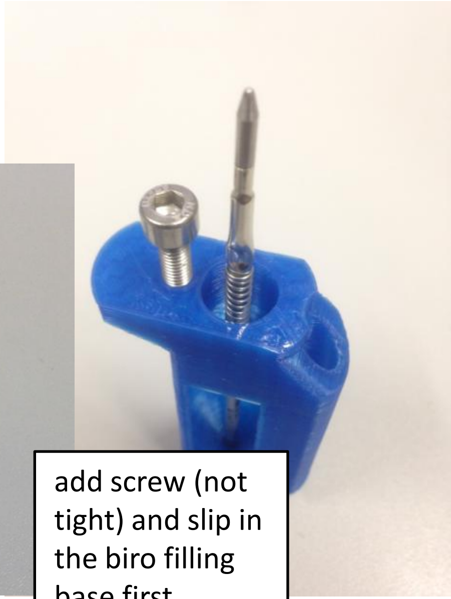

add screw (not tight) and slip in the biro filling base first

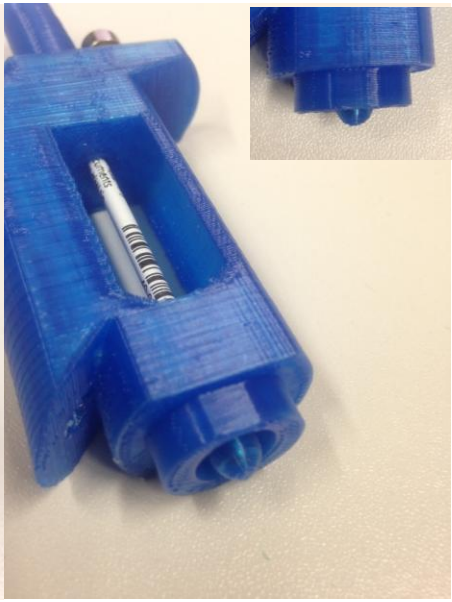

Note 1: part C may need a tad glue if it doesn‘t stick to the biro filling by itself.

Note 2: If the spring mechanism is too weak, add something (e.g. A washer) beneath the spring. The springy range should be at least as great as your part E height (Range Z, default = 10 mm)

Assembly 3/5

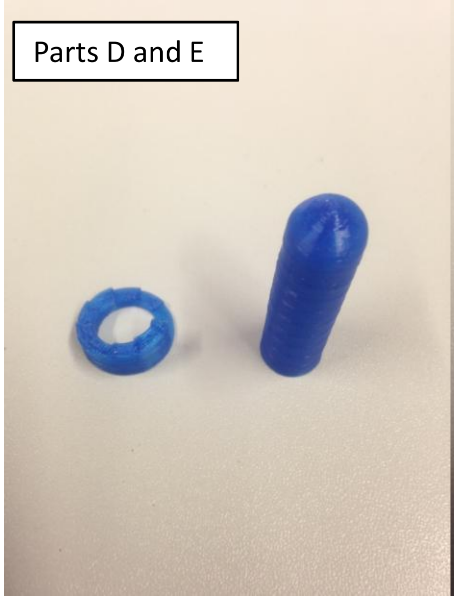

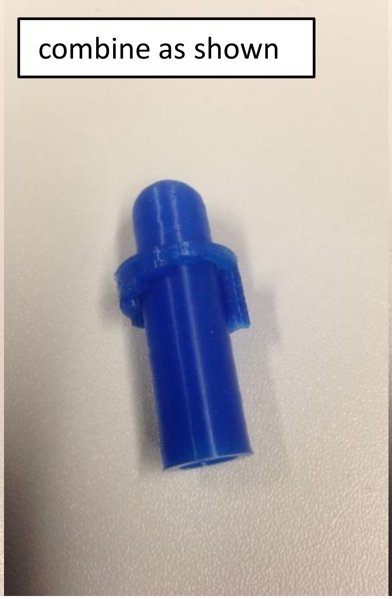

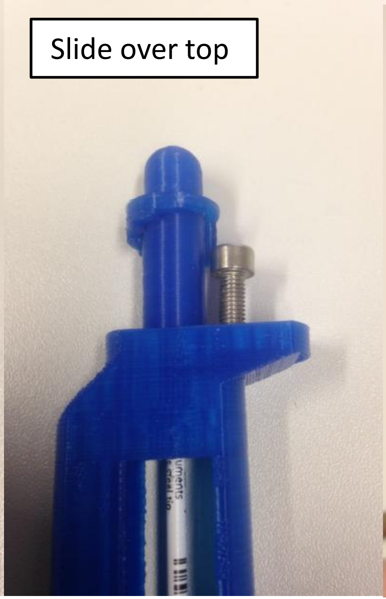

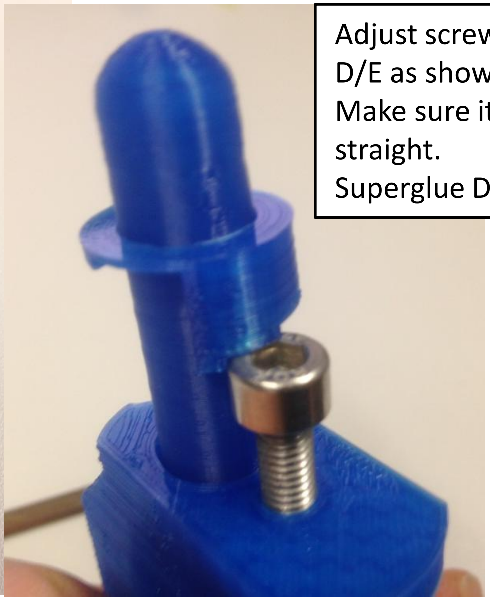

Adjust screw and D/E as shown. Make sure it is straight. Superglue D/E.

Note: part D may need a tad glue if it doesn‘t stick to the biro filling by itself

Assembly 4/5

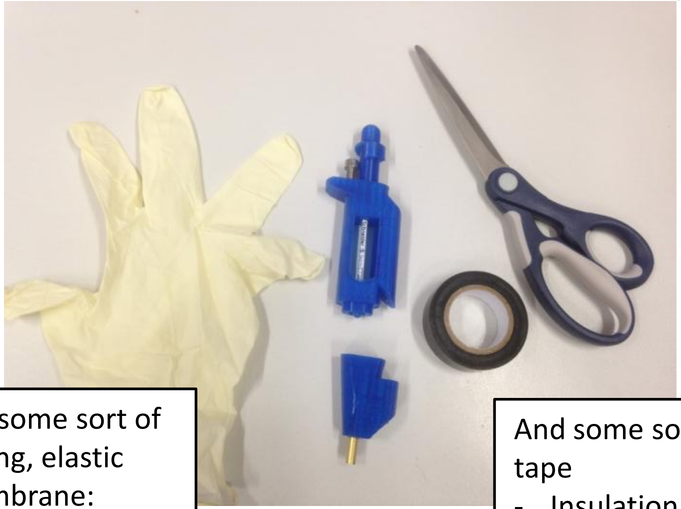

Get some sort of strong, elastic membrane:

- Lab glove
- Baloon
- Condom
- ...

And some sort of tape

- Insulation tape
- Parafilm
- Gaffa?
- ...

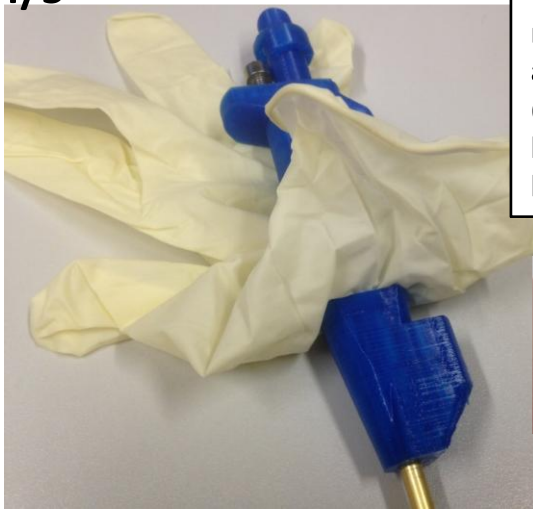

Make sure the membrane is stretched a tad over the top part (part C should bulge a bit), then slide over the bottom part.

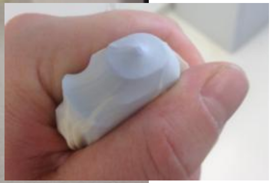

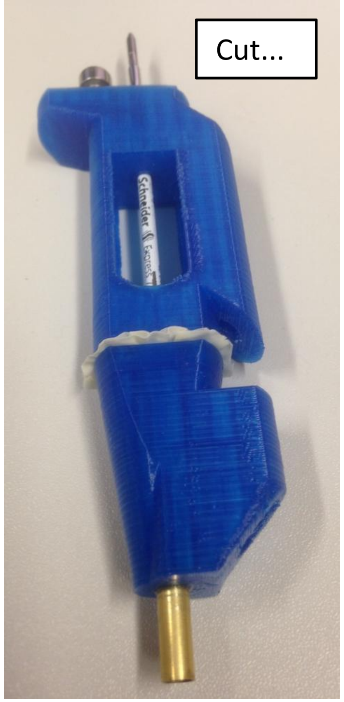

Cut...

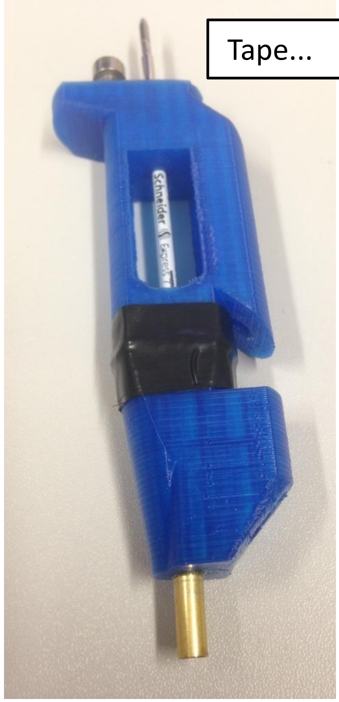

Tape...

Assembly 5/5

Parts F and G

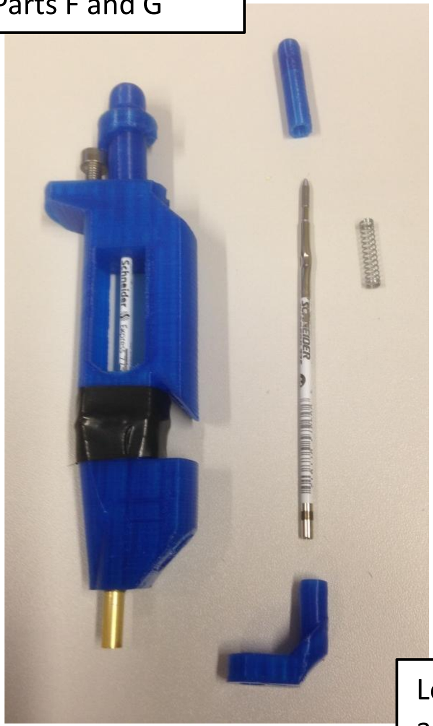

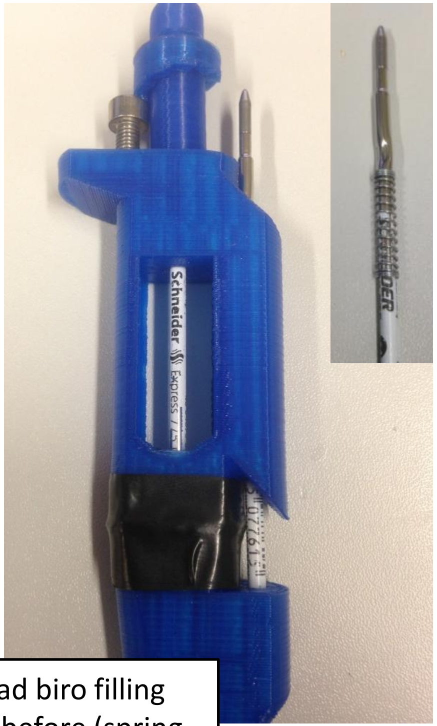

Load biro filling as before (spring on wrong side, and add a washer or something if it isn‘t „springy“

Slip over part F (glue if needed)

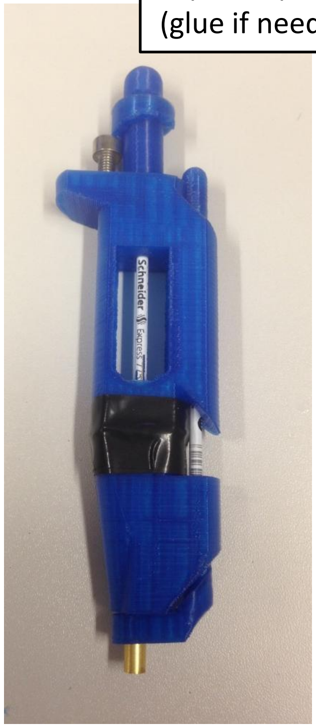

Add part G (glue if needed)

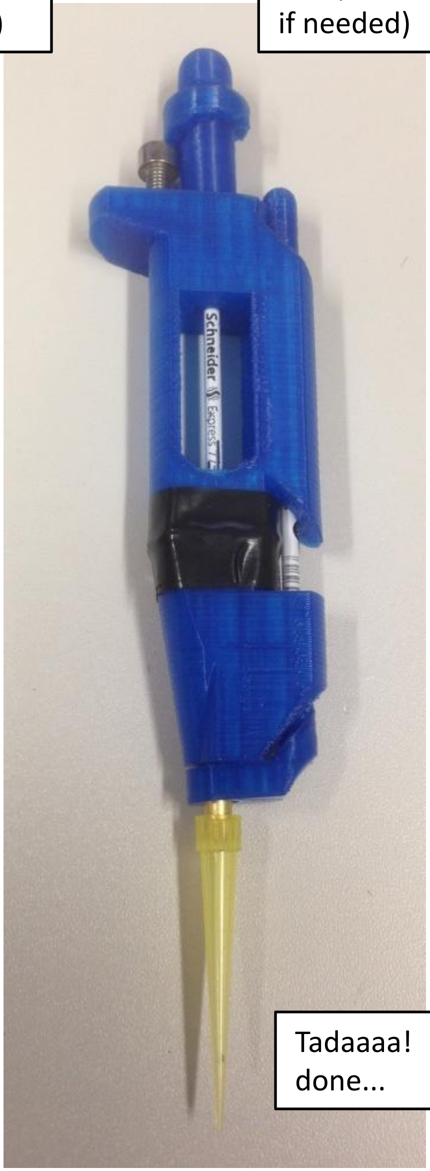

Tadaaaa! done...

Note: if something doesnt slide nicely, ream it with a dremel, and if that doesnt help, try greasing it as well. Also, greasing the biro fillings may help... If everything went well, the pipette should feel stable and not wobbly/jiggly... If too jiggly, try to print with lower “tolerance“ setting (customizer). If nothing moves, increase tolerance.

Some different makes

Some prototypes...

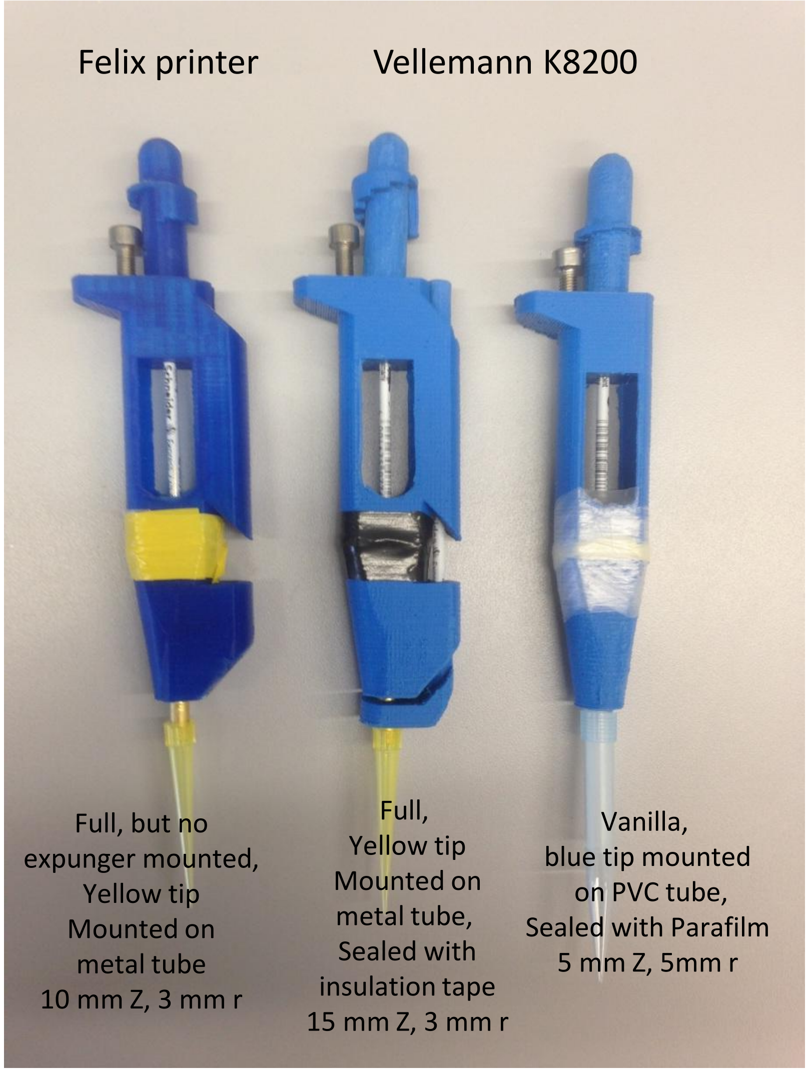

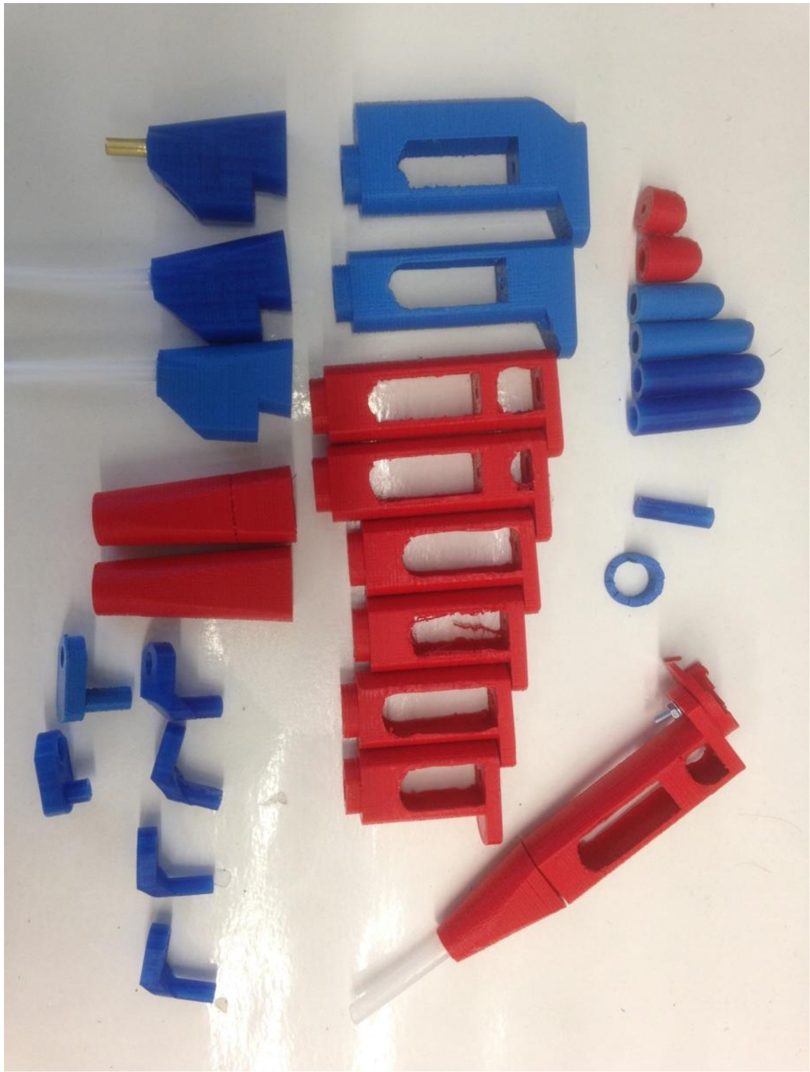

Quick and dirty “weight“ calibration of 2 different makes...

both roughly equate a P200 and are quite linear

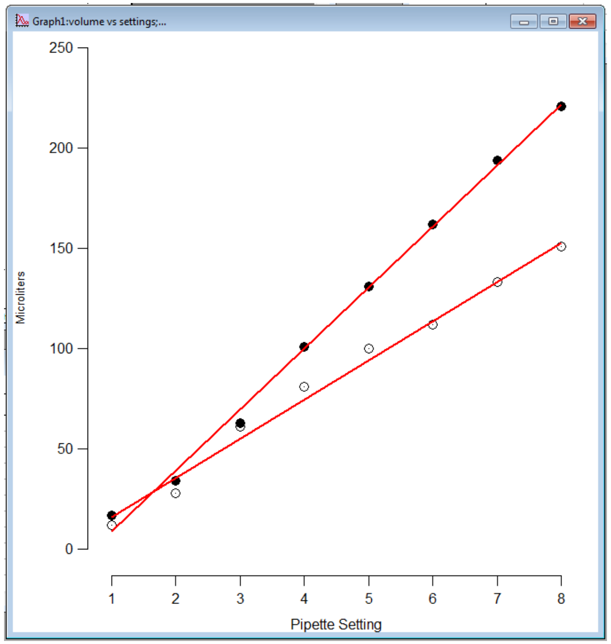

Science in Africa NGO

TReND in Africa gUG: http://trendinafrica.org/

Help us design cheap, good lab equipment!

http://trendinafrica.org/activities/open-source/open-source-main/
