# Supplementary material for: Open Labware: 3-D Printing Your Own Lab Equipment
Source: PLoS Biol. 2015 Mar 20;13(3):e1002086. doi: 10.1371/journal.pbio.1002086 (PMC4368627; doi:10.1371/journal.pbio.1002086)

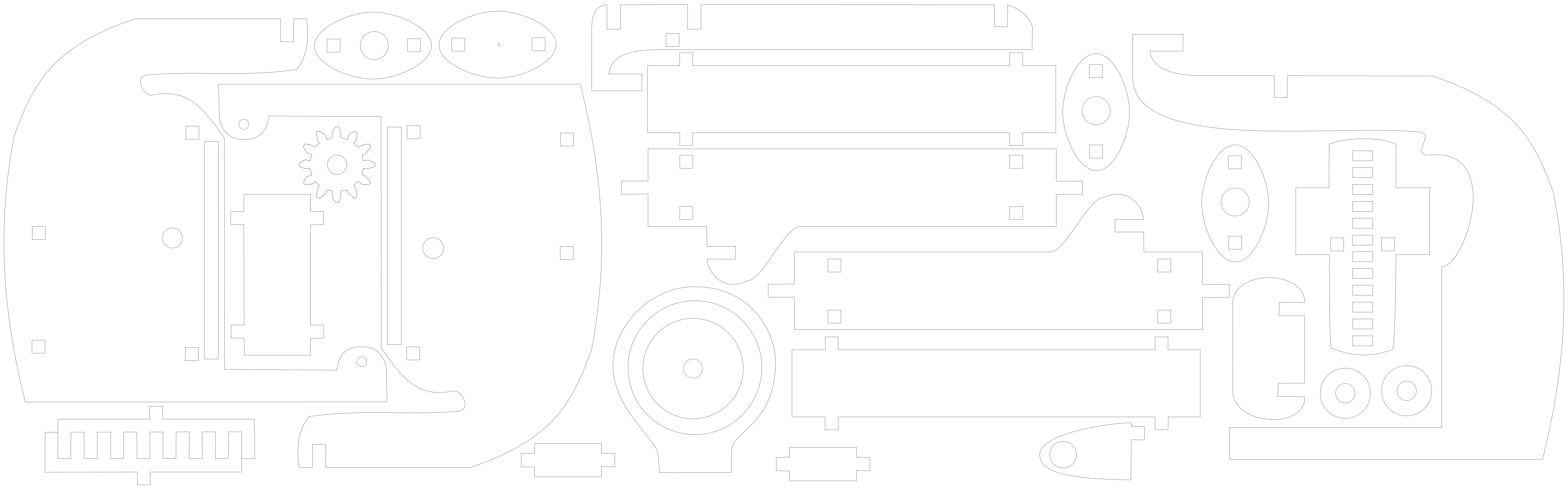

Copyright Zombie Cat, Inc.

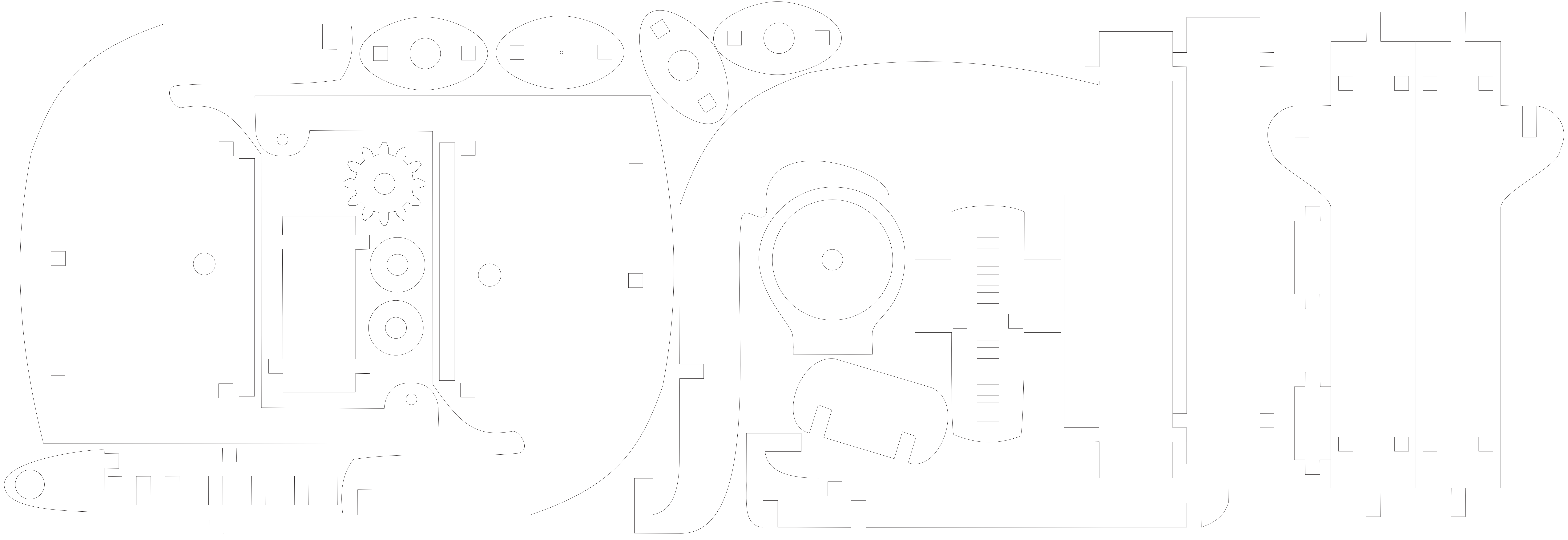

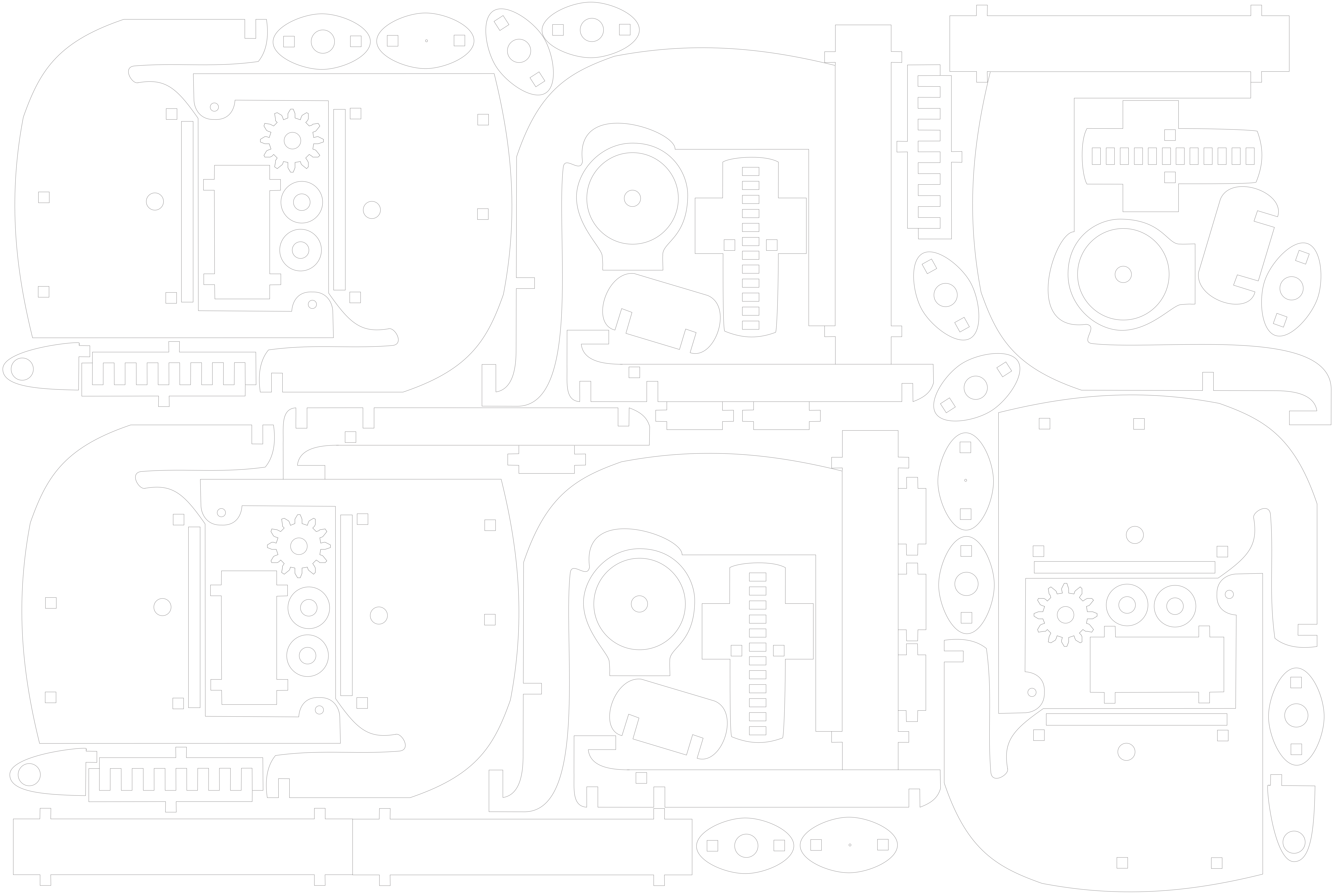

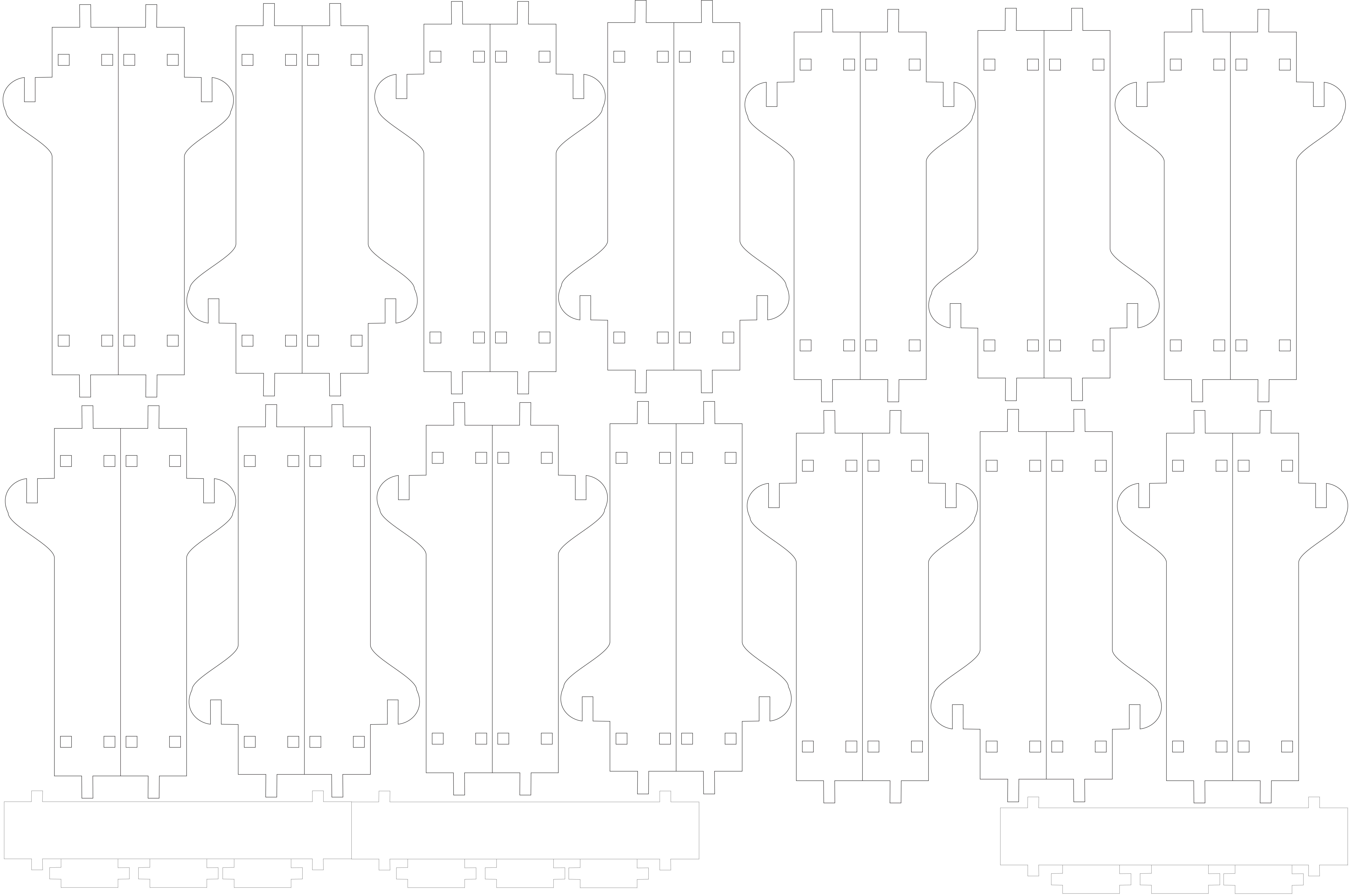

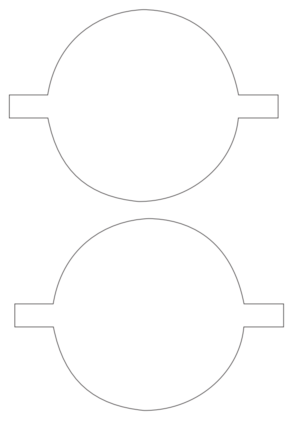

Supplement: S1 Data — Container with all files mentioned in the article that are currently hosted on thingiverse (www.thingiverse.com). (ZIP) [file pbio.1002086.s001.zip › Microscope_with_iPad_attachement - by ZombieCat.zip!/Microscope_with_iPad_attachment/Laser_cut_microscope.pdf]
