# Supplementary material for: Open Labware: 3-D Printing Your Own Lab Equipment
Source: PLoS Biol. 2015 Mar 20;13(3):e1002086. doi: 10.1371/journal.pbio.1002086 (PMC4368627; doi:10.1371/journal.pbio.1002086)
Supplement: S1 Data — Container with all files mentioned in the article that are currently hosted on thingiverse (www.thingiverse.com). (ZIP) [file pbio.1002086.s001.zip › OpenFuge - by CopabX.zip!/OpenFuge/Schematic_Prints_v1.8.pdf]

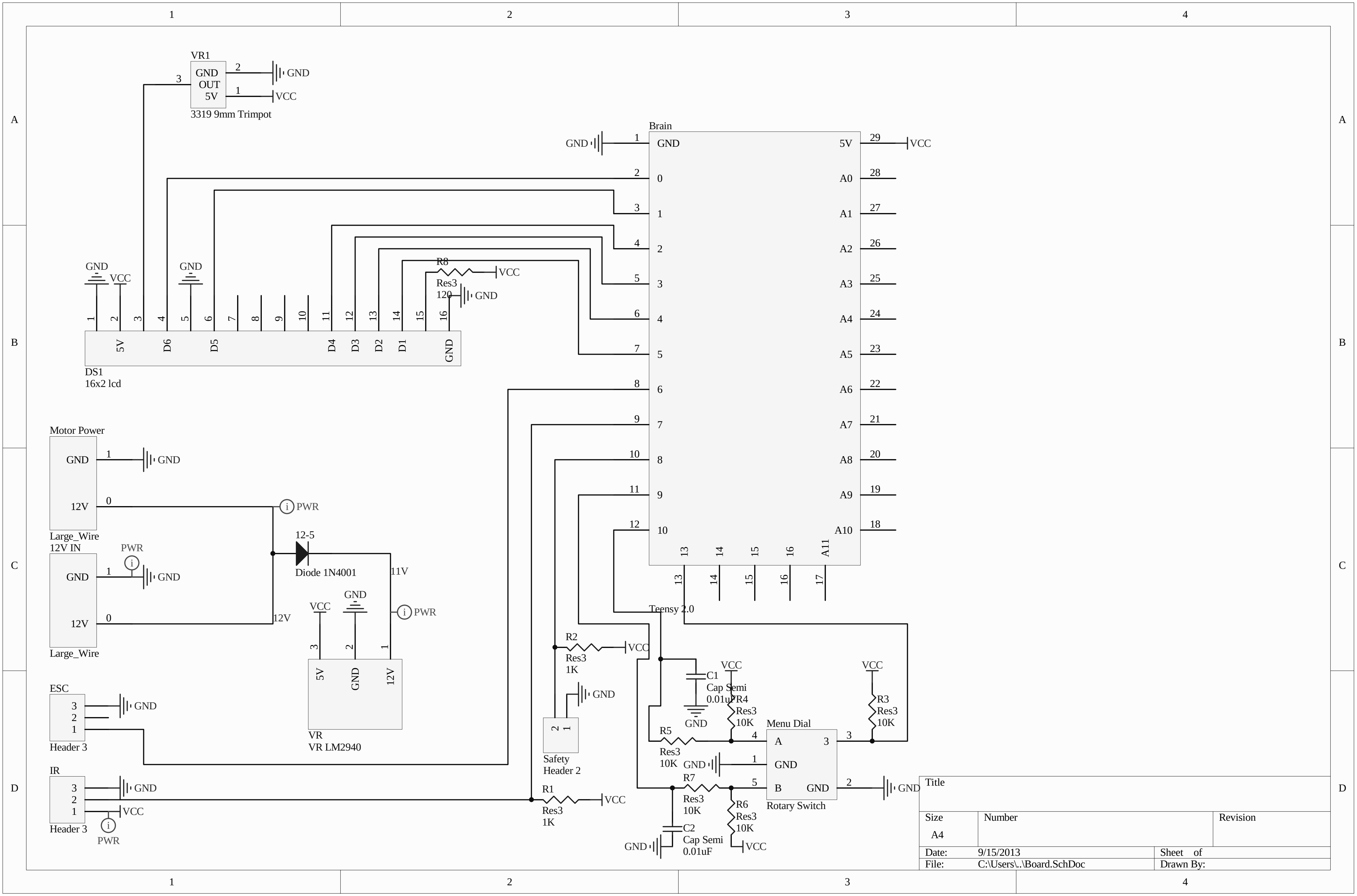
VR1
GND
OUT
5V
3319 9mm Trimpot
GND
VCC
Brain
GND
5V
0
1
2
3
4
5
6
7
8
9
10
A0
A1
A2
A3
A4
A5
A6
A7
A8
A9
A10
A11
Teensy 2.0
R8
Res3
120
DS1
16x2 lcd
D6
D5
D4
D3
D2
D1
Motor Power
Large_Wire
12V IN
Large_Wire
12V
PWR
12-5
Diode 1N4001
11V
VR
VR LM2940
ESC
Header 3
IR
Header 3
R1
Res3
1K
R2
Res3
1K
Safety
Header 2
C1
Cap Semi
0.01uF
R4
Res3
10K
R3
Res3
10K
R5
Res3
10K
R7
Res3
10K
R6
Res3
10K
C2
Cap Semi
0.01uF
Menu Dial
Rotary Switch
Title
Size
A4
Number
Revision
Date:
9/15/2013
File:
C:\Users\..\Board.SchDoc
Sheet of
Drawn By:
