# Supplementary material for: Open Labware: 3-D Printing Your Own Lab Equipment
Source: PLoS Biol. 2015 Mar 20;13(3):e1002086. doi: 10.1371/journal.pbio.1002086 (PMC4368627; doi:10.1371/journal.pbio.1002086)
Supplement: S1 Data — Container with all files mentioned in the article that are currently hosted on thingiverse (www.thingiverse.com). (ZIP) [file pbio.1002086.s001.zip › Parametric_3_axis_manipulator_-_optional_servo_mounts - by T Baden.zip!/Parametric_3_axis_manipulator_-_optional_servo_mounts/Manipulator_assembly_Instructions.pdf]

Parametric 3 axis manipulator with tiltable electrode holder and optional servo mounts*

Written in OpenSCAD by Tom Baden, Centre for Integrative Neuroscience (CIN), University of Tübingen, Germany
(Thomas.Baden@uni-tuebingen.de; www.tombaden.wordpress.com)

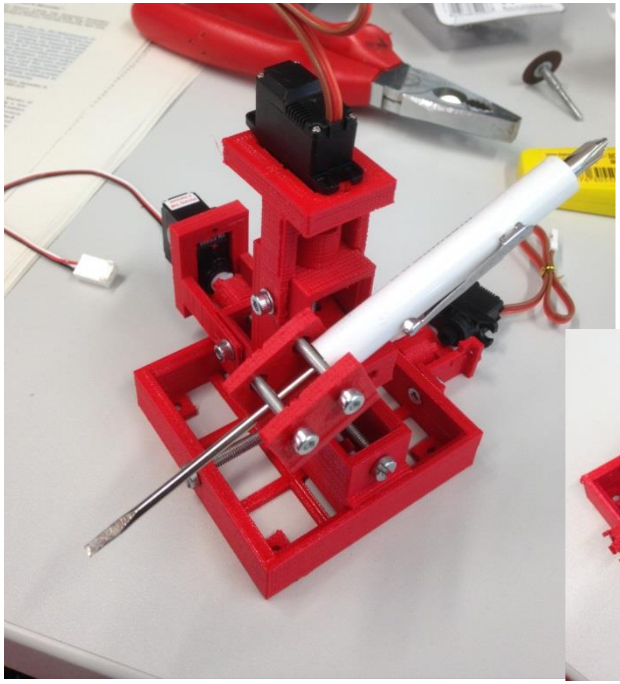

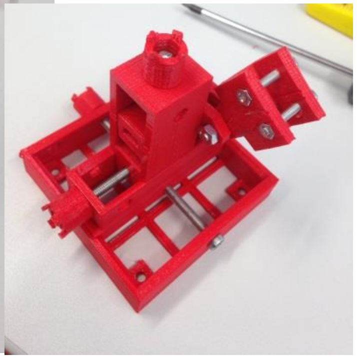

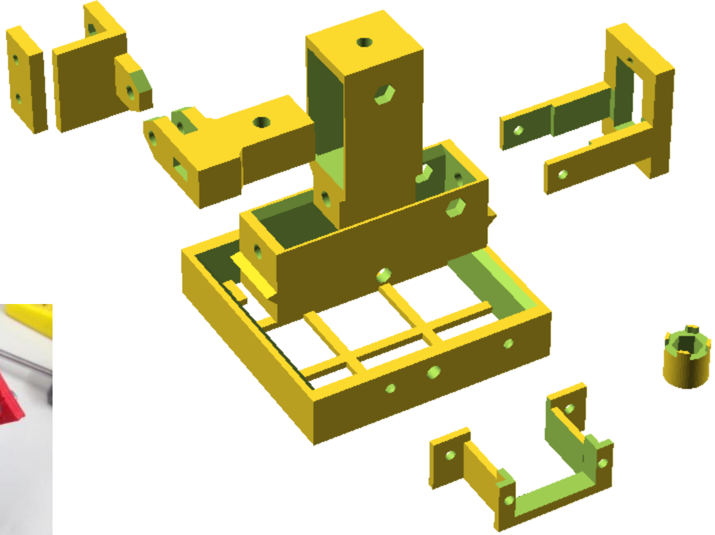

*inspired by the Backyard Brains „Searcher“ Model by Tim Marzullo
(https://backyardbrains.com/products/micromanipulator)

The main difference between this model and the original (aside from minor modifications)

1) The presence of a real z axis (the Searcher z axis is slanted 45 degrees)
2) Option to add continuous rotation micro-servo motors
3) Implementation in OpenSCAD for easy modification / customizer compatability

printed on Vellemann K8200
PLA; z = 0.35 mm; no supports

Overview of printed parts

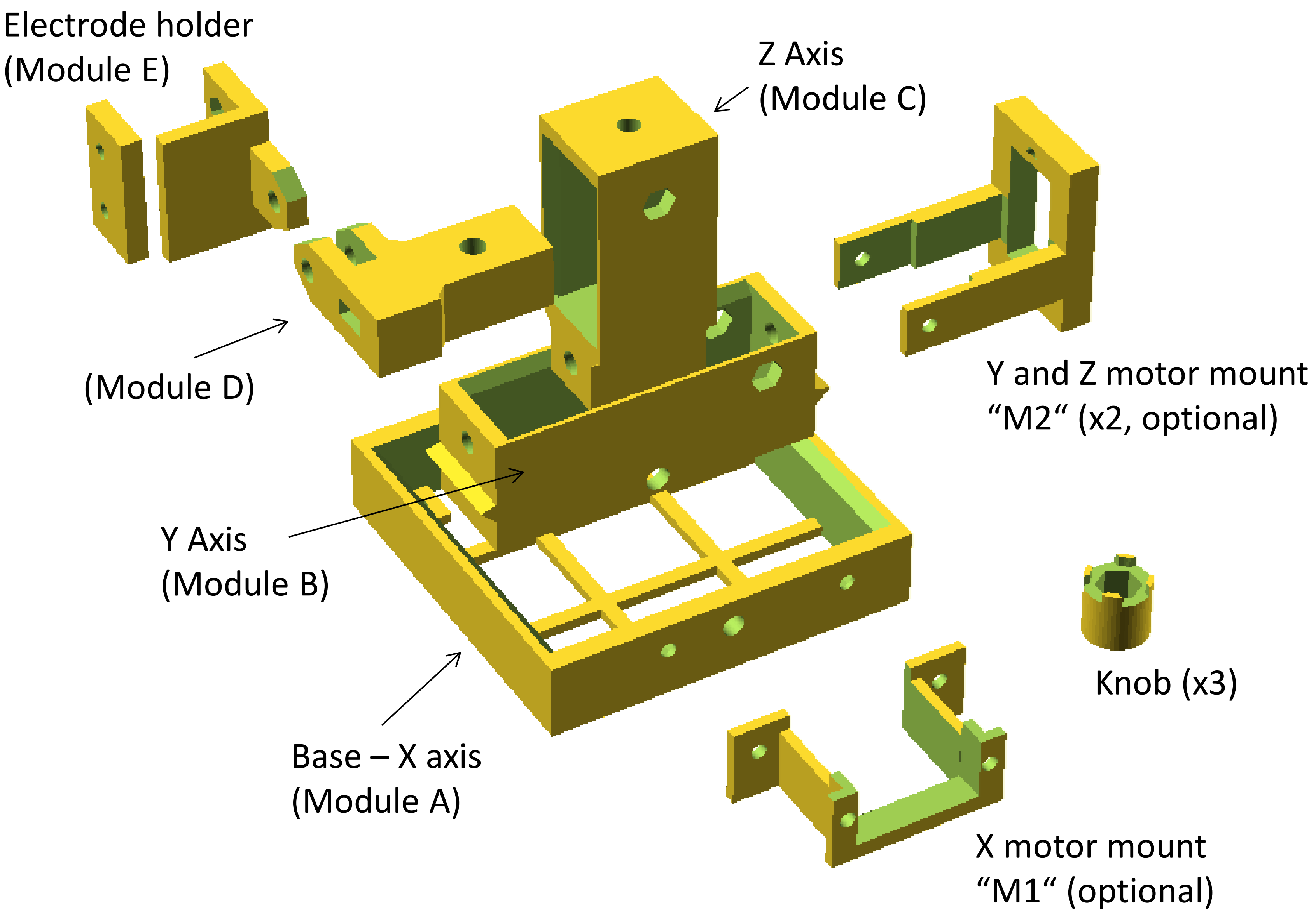

List of parts

1) All unique printed parts x1, except for knobs (x3) and M2 mount (x2)

2) Screws:
    - 4* M4 (80, 75, 50 and 30 mm)
    - 2* M3 (25 mm)
    - 6* M3 (6 mm) – optional, for motor mounts

3) Boats:
    - 6* M4 (normal)
    - 3* M4 (closing ones)
    - 2* M3 (normal)
    - 6* M3 (normal) – optional, for motor mounts
    - 6* M3 washers – optional, for motor mounts

4) Dremel, grease!, screwdriver, pliars,

5) Superglue (optional)

6) 3* Continuous rotation micro-servos (optional)

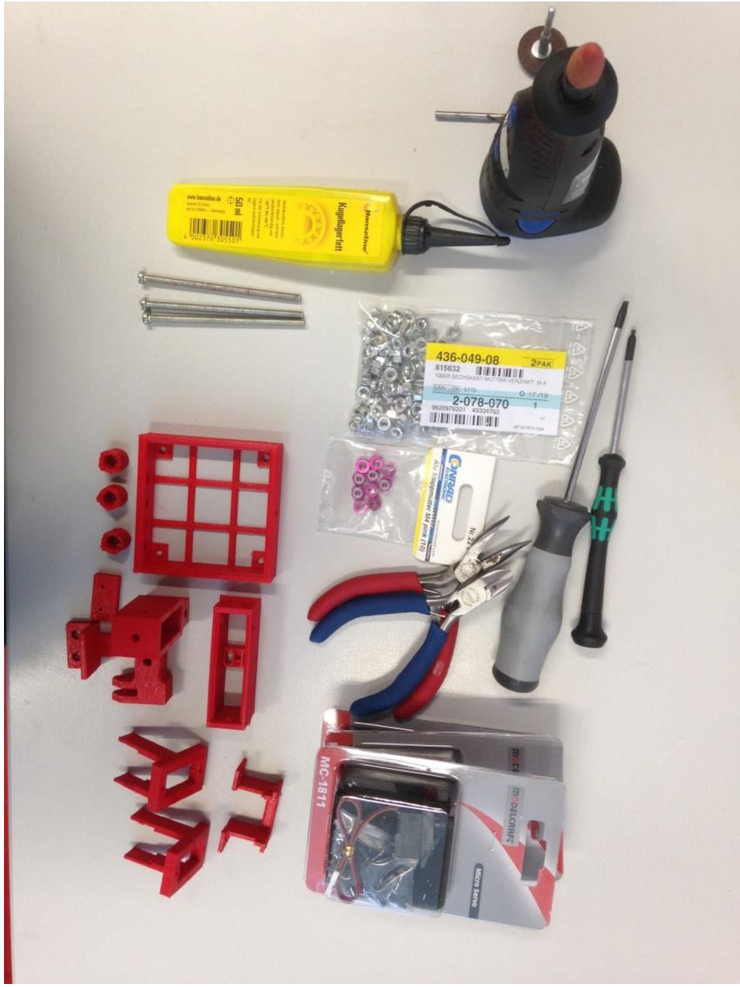

Step 1: Grease everything that moves

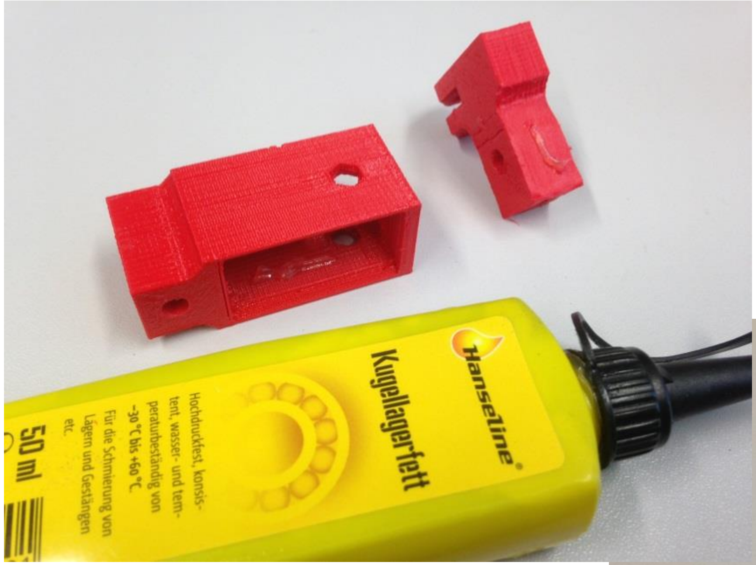

- All sliding parts
- The 3 main screws

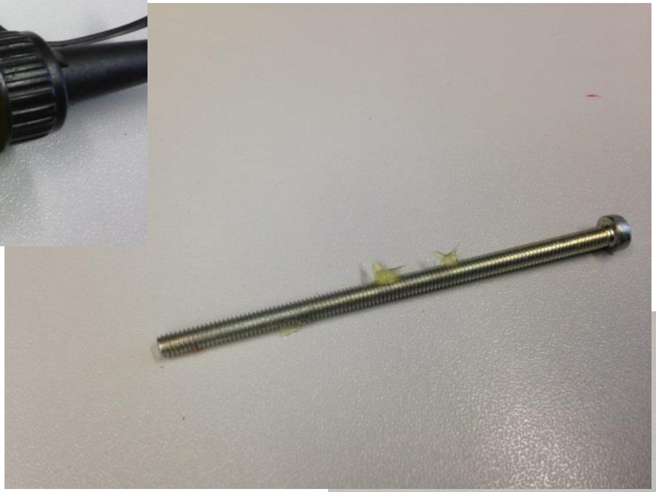

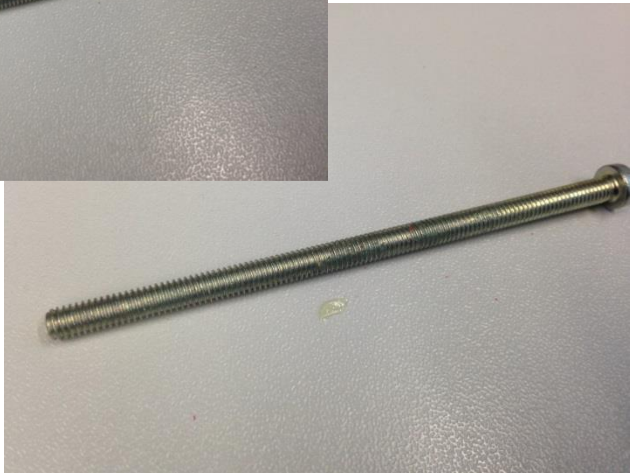

Also, clean all poorly printed parts (sand down / ream)
Everything should slide smoothly when put together

Step 2: Mounting Module D onto C

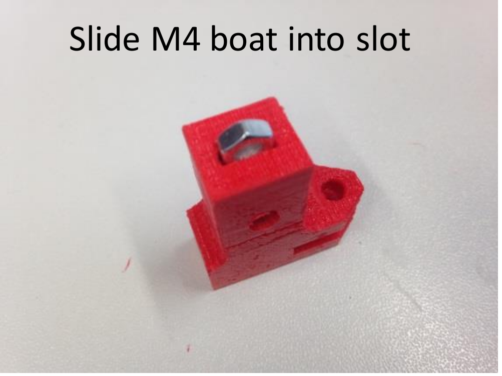

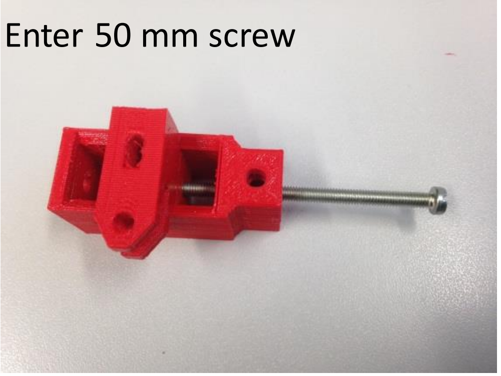

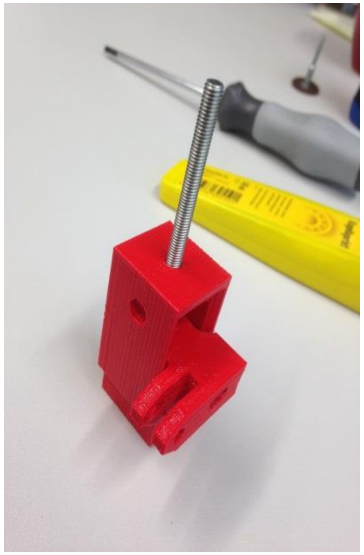

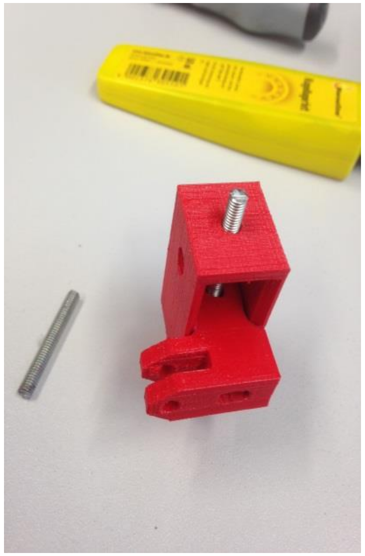

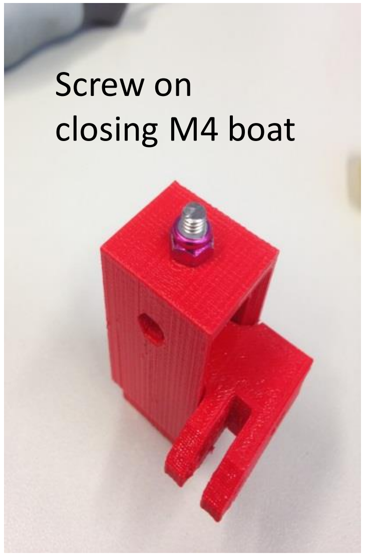

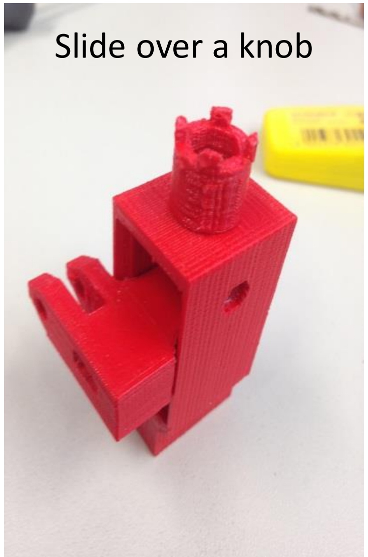

If it sticks out the top (here I used an 80 mm screw) – cut it off to a few mm above the top

Turning the knob should be easy, and smoothly move the electrode link (D) up and down.
Optional: with D perfectly in place, superglue the M4 boat inside Module D into place

Step 3: Mounting Module C/D onto Module B

Slide 2* M4
boats into slots

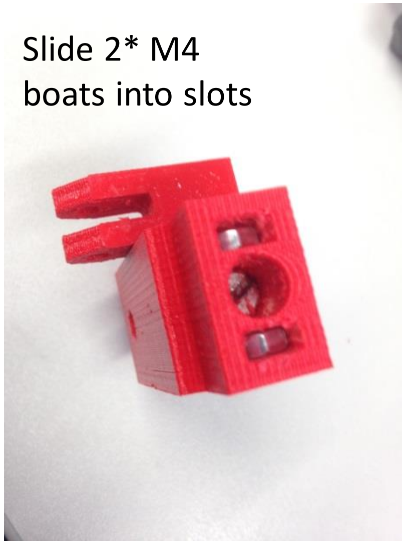

Cut screw if necessary

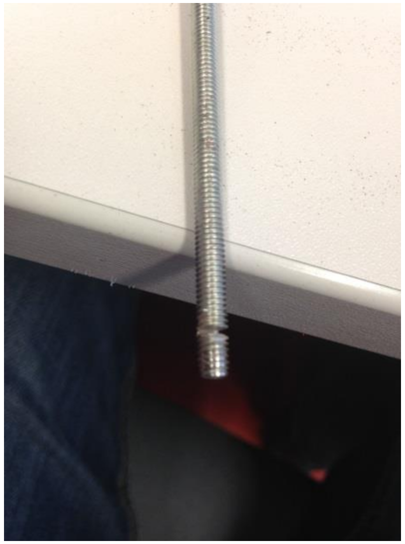

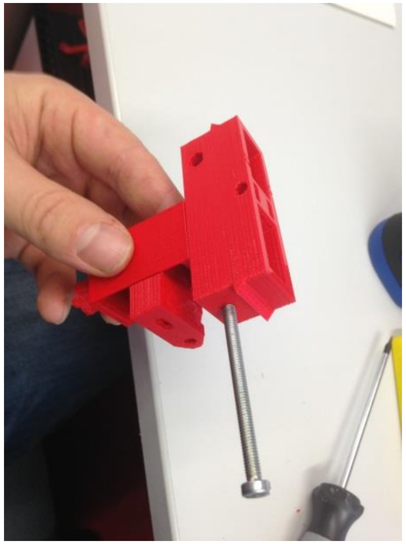

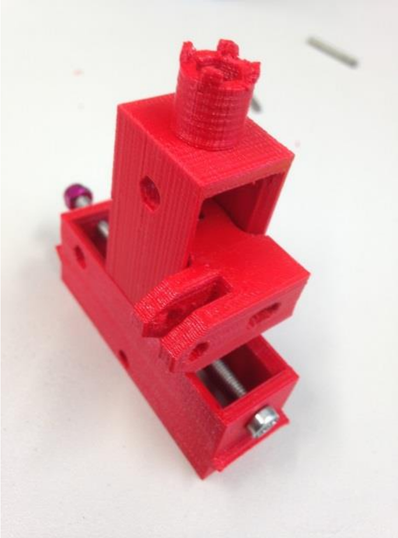

Enter screw and fix with closing M4 boat

Step 4: Mounting Module B/C/D onto Module A

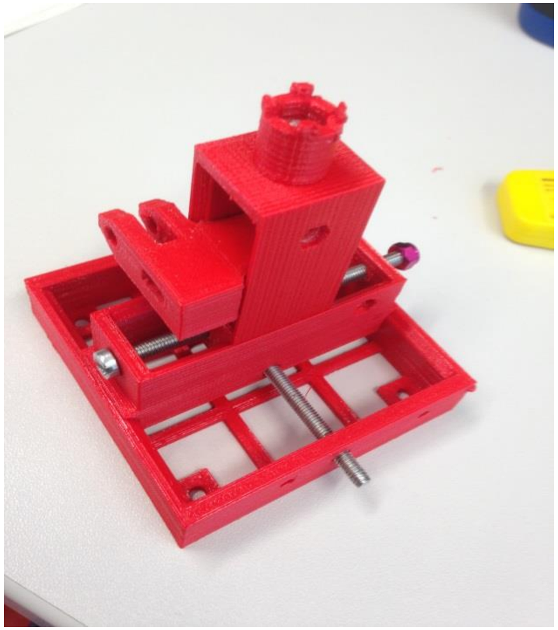

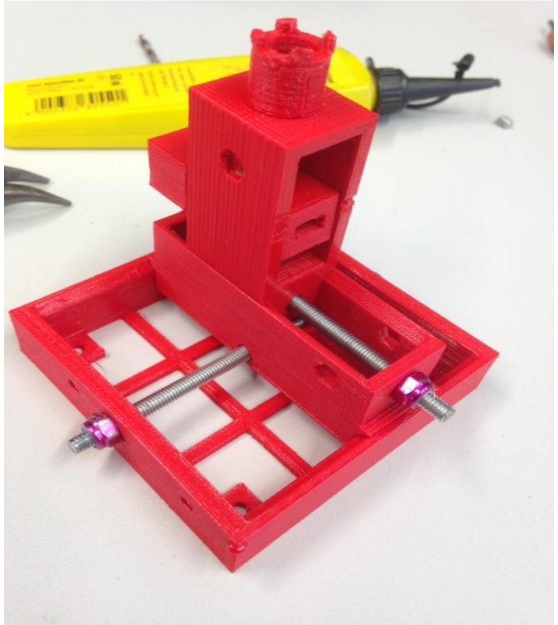

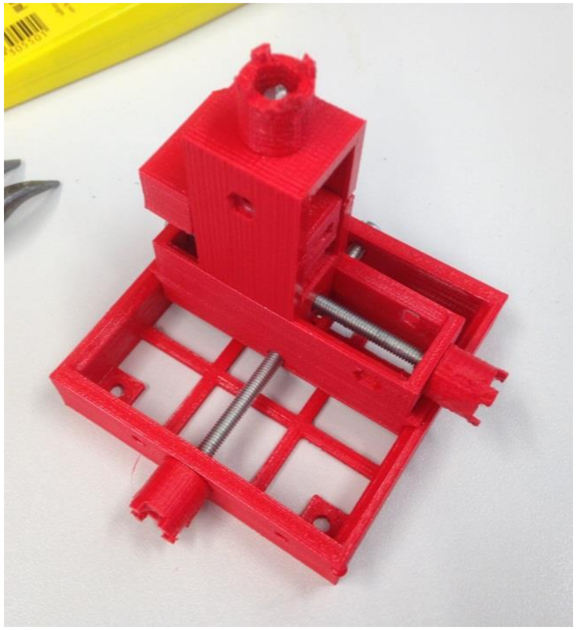

As before, enter 2* M4 boats into bottom of B, feed through screw and fix with closing M4 boat

Slide over the knobs

All 3 axes should now move smoothly when the respective knob is turned.

Step 5: Putting together the electrode holder

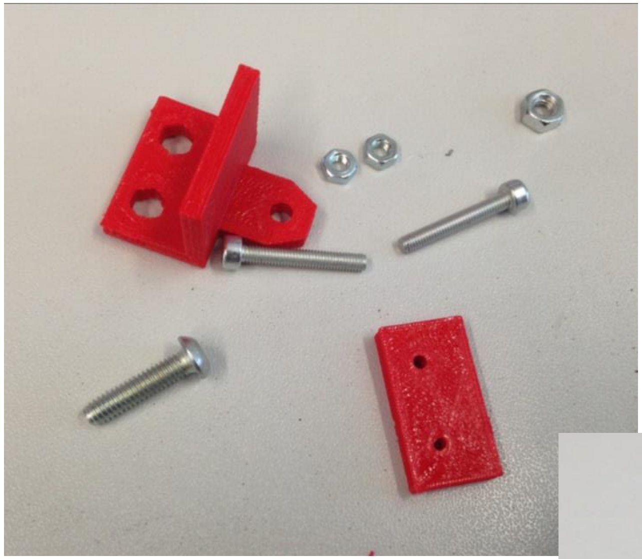

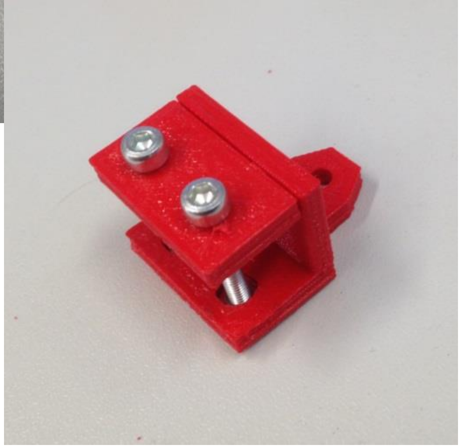

Slot 2 M3 boats into the holder, ream the srewholes if necessary and assemble

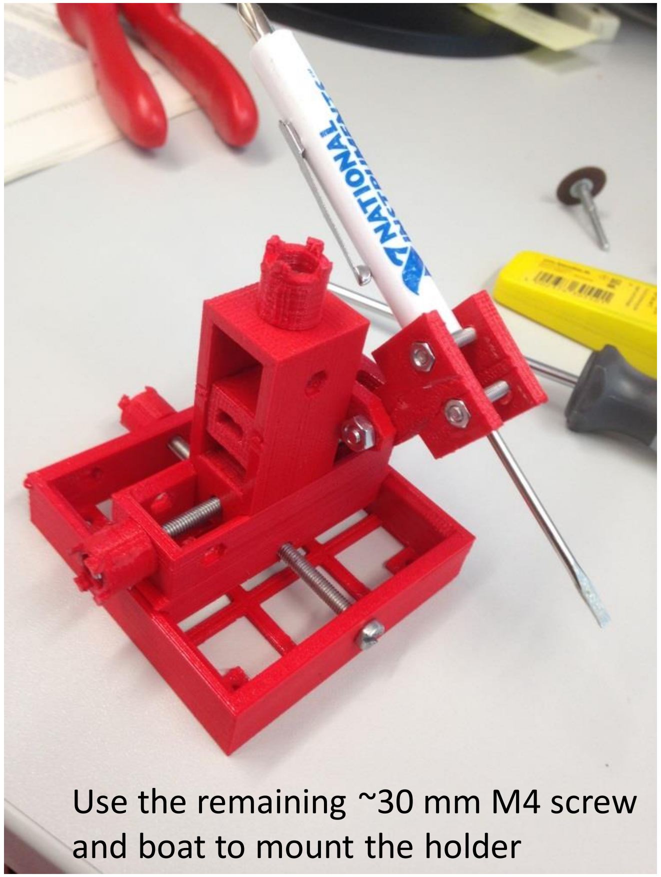

Use the remaining ~30 mm M4 screw and boat to mount the holder

If you choose to not add motors, this is the finished (manual) manipulator

Step 6: Adding optional micro-servo mounts

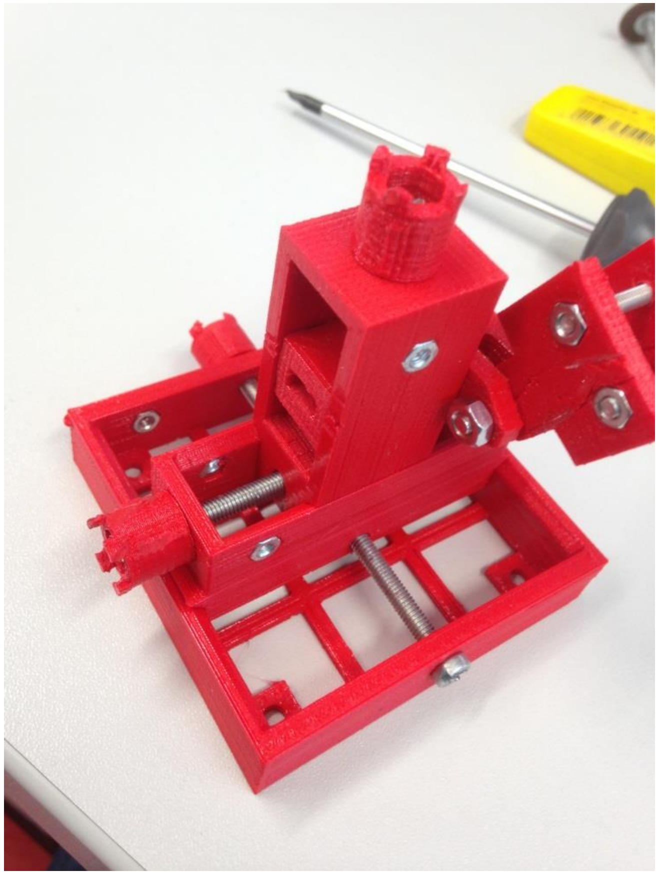

Enter 6 M3 boats into the 6 slots (2 on A, B and C, each)

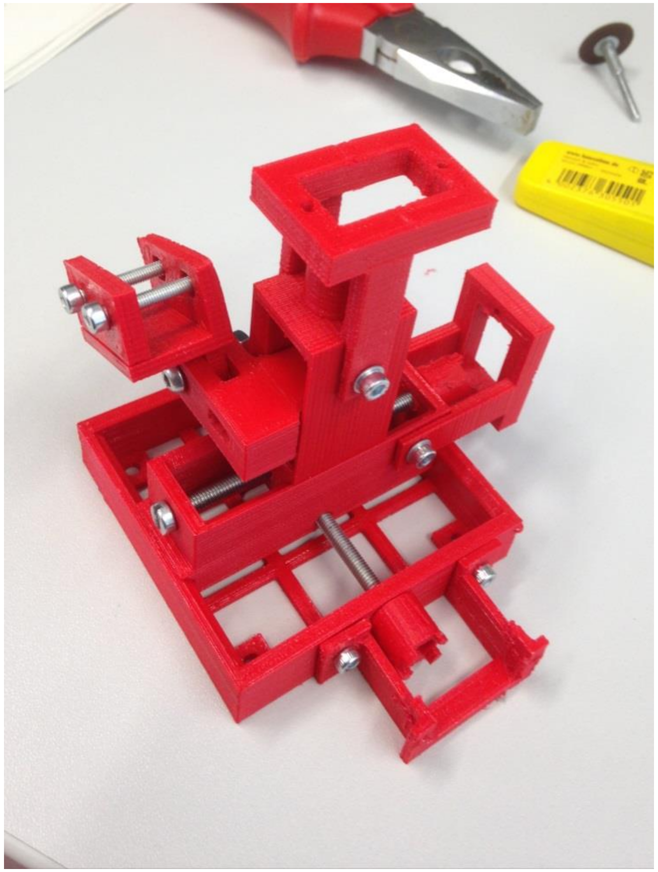

Screw on the motor holders, use washers if the screw sticks out on the inside

Final: Add the motors

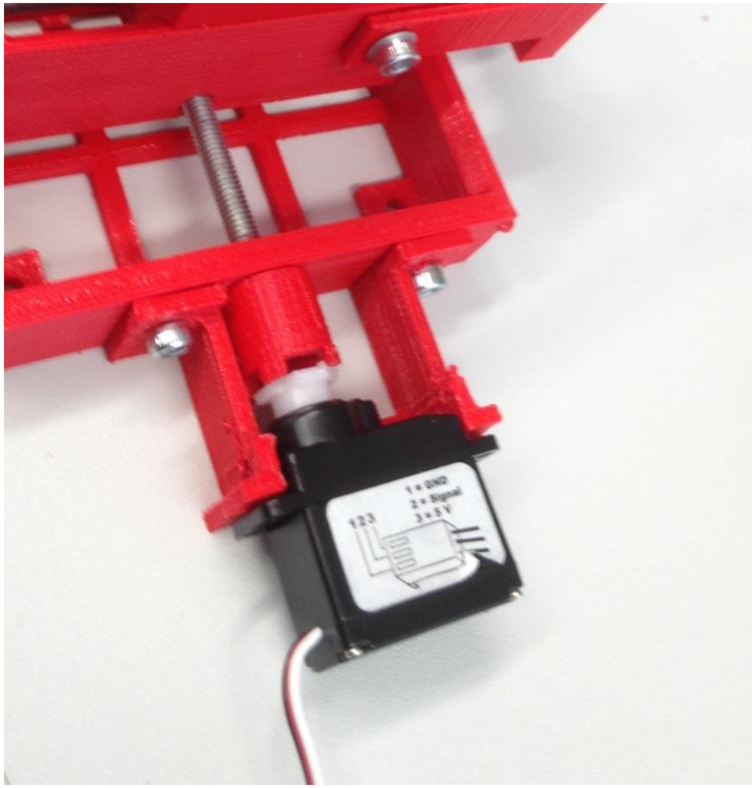

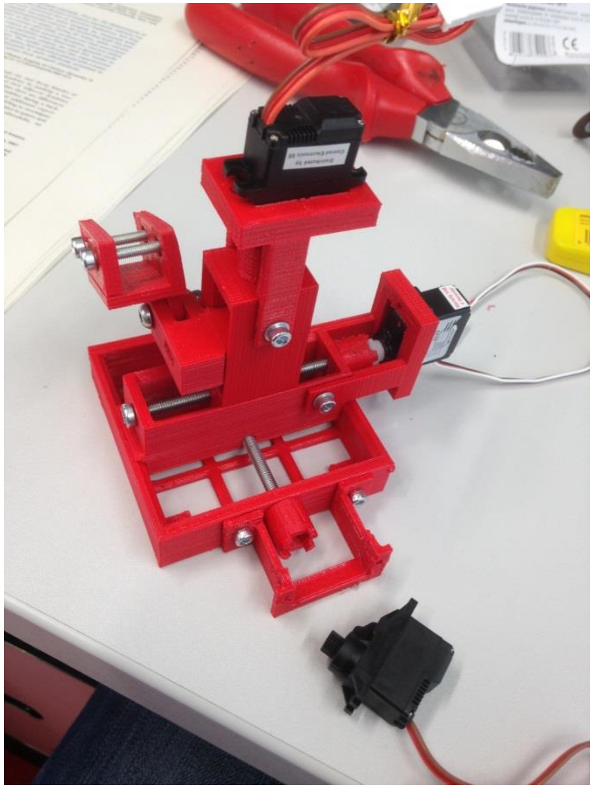

Put on the 3 micro servos. Take the cross-shaped motor clip which comes with the servo and cut all 4 wings almost (!) at the base. These should fit into the grooves of the knobs. Screw motors on with appropriate screws (usually small, sharp ones).
If motors don‘t grip nicely, add another M4 boat onto the axis and slide up the knob (or print a longer knob)
