# Supplementary material for: Open Labware: 3-D Printing Your Own Lab Equipment
Source: PLoS Biol. 2015 Mar 20;13(3):e1002086. doi: 10.1371/journal.pbio.1002086 (PMC4368627; doi:10.1371/journal.pbio.1002086)
Supplement: S1 Data — Container with all files mentioned in the article that are currently hosted on thingiverse (www.thingiverse.com). (ZIP) [file pbio.1002086.s001.zip › Parametric_3_axis_manipulator_-_optional_servo_mounts - by T Baden.zip!/Parametric_3_axis_manipulator_-_optional_servo_mounts/Motorising_the_manipulator.pdf]

Motorising the manipulator with continuous rotation micro-servos and Arduino

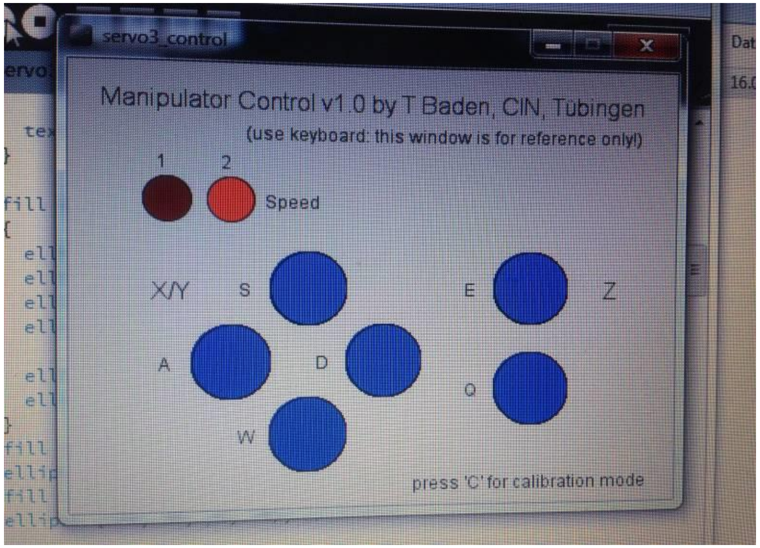

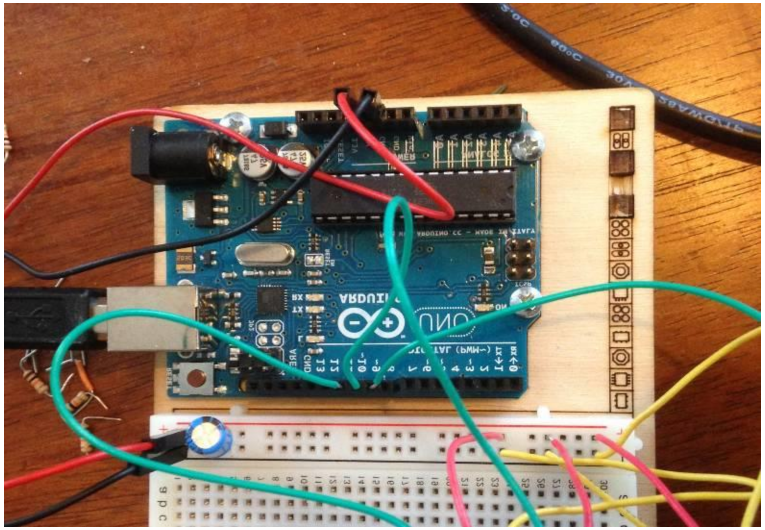

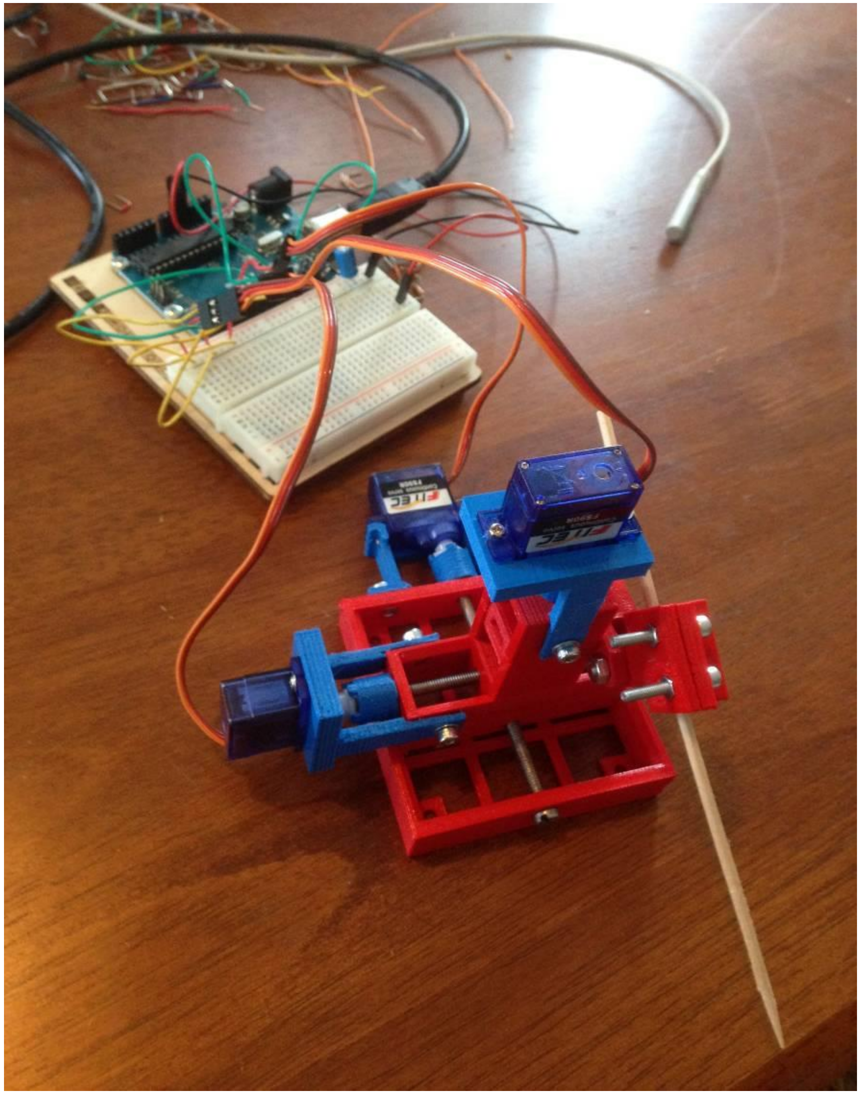

Hardware side:

1) Connect power and ground of the 3 servos to 0 vs 5 V power supply. You can use the ones on the Arduino board (“5V“ and “GND“, use a discharge capacitor in the circuit!) but then you run the risk of frying your Arduino / USB port / motherboard if the servos draw too much power (e.g. when stuck). So for safety, find another 5V power supply. Dont use a 9V battery. That will fry the motors (unless you use a step down). Depending on the tolerance of your servos, 6 V might work (i.e. 4 AA batteries, untested).

2) Connect the control pins of each motor to the PWM ports of the Arduino. In my code I defined:

Analog out pin 11 (PWM) – X Axis servo
Analog out pin 10 (PWM) – Y Axis servo
Analog out pin 9 (PWM) – Z Axis servo

Software side:

1) Through the arduino software, upload the “Servo3“ file to your arduino.

2) Install “Processing“: http://processing.org/

3) Load the file “Servo3_control“ in processing

4) Make sure my code addresses the correct serial port. For this, look for this line in the code:

```
String portName = Serial.list()[0];
```

and change the “0“ to 1, 2, 3, 4.. as appropriate. (usually, 0 is fine though, so try that 1st if in doubt)

5) Run the Processing script (play button in top of window). On your screen you should now see the control window as shown on the 1st page of this document. (almost there!). I hope the controls are self explanatory.

Calibration and stuff

Quite possibly, your servos are going to “hum“ a bit, or perhaps even turn at a low speed. No worries, this is normal. Basically this happens as continuous rotation servos are not perfectly calibrated to an input signal of 90 degrees meaning “no rotation“ – they can be off by a few degrees. There are 2 ways of fixing this:

- Quick and dirty: Use the “calibration“ option in my code. Press “c“ – the buttons on the screen should turn light blue. Now press any of the control buttons that normally move the motor (A,S,W,D,Q,E). Now, instead of moving the servos, pressing the button will change the “zero“ calibration“ of this motor by plus / minus 1. The programm will jump back immediately to normal control mode (dark blue buttons). Repeat until all motors stop moving / humming. Annoyingly, you‘d have to do this every time you connect. So:

- Update the arduino code. When you calibrate useing the above procedure, it will give the new “zero“ angle in the bottom (in processing). This should be a number close to 90 for each axis. You want to enter this number, as calibrated for your servos, into the arduino code. Open the code (Servo 3), and find the variables.

  ```
  int Xzero_angle = 93;        int Yzero_angle = 94;   int Zzero_angle = 90;
  ```

  Note that most are not 90. These are the calibration values needed for the 3 servos i happen to use. Just change these as appropriate, and upload to the Arduino.Now, all should be working.

Disclaimer:

I know that my code is not particularly elegant, nor clean. I am new to Arduino and Processing, and I did not want to spend too much time on this. Suggestions for improvements always welcome.
